# Predicting Hybrid Performances for Quality Traits through Genomic-Assisted Approaches in Central European Wheat

**DOI:** 10.1371/journal.pone.0158635

**Published:** 2016-07-06

**Authors:** Guozheng Liu, Yusheng Zhao, Manje Gowda, C. Friedrich H. Longin, Jochen C. Reif, Michael F. Mette

**Affiliations:** 1 Department of Breeding Research, Leibniz Institute of Plant Genetics and Crop Plant Research (IPK), Stadt Seeland, Germany; 2 State Plant Breeding Institute, University of Hohenheim, Stuttgart, Germany; University of Guelph, CANADA

## Abstract

Bread-making quality traits are central targets for wheat breeding. The objectives of our study were to (1) examine the presence of major effect QTLs for quality traits in a Central European elite wheat population, (2) explore the optimal strategy for predicting the hybrid performance for wheat quality traits, and (3) investigate the effects of marker density and the composition and size of the training population on the accuracy of prediction of hybrid performance. In total 135 inbred lines of Central European bread wheat (*Triticum aestivum* L.) and 1,604 hybrids derived from them were evaluated for seven quality traits in up to six environments. The 135 parental lines were genotyped using a 90k single-nucleotide polymorphism array. Genome-wide association mapping initially suggested presence of several quantitative trait loci (QTLs), but cross-validation rather indicated the absence of major effect QTLs for all quality traits except of 1000-kernel weight. Genomic selection substantially outperformed marker-assisted selection in predicting hybrid performance. A resampling study revealed that increasing the effective population size in the estimation set of hybrids is relevant to boost the accuracy of prediction for an unrelated test population.

## Introduction

Bread wheat (*Triticum aestivum* L.) is one of the most important crops grown on 200 million hectares of farmland worldwide [1] and the development of varieties with good bread-making quality has been a major target in wheat breeding. The bread-making quality of wheat is a complex property controlled by both genetic background and environmental conditions [2]. The bread-making properties of wheat flour are mainly determined by relative content and features of gluten proteins [3], which are quantified in breeding programs using the sedimentation (SDS) test according to Zeleny. Similarly, total protein content is an important indicator of baking quality and nutritional value of wheat. Test weight and 1000-kernel weight are relevant for flour millers who use these traits to describe wheat kernel composition and potential flour extraction [4]. Measuring quality traits in wheat is often labor-intensive. Therefore, quality traits are interesting targets for the application of genomic-assisted crop improvement.

Genomic-assisted crop improvement can either be based on marker-assisted selection [5] or genomic selection [6]. In marker-assisted selection, genotypic values of individuals are predicted based on the effects of a limited number of selected markers. In contrast, genomic selection considers an extensive number of markers without performing marker-specific significance tests [7–9]. The comparative efficiencies of marker-assisted versus genomic selection depend on the genetic architecture underlying the respective traits under consideration. Marker-assisted selection is most effective for traits influenced by a few quantitative trait loci (QTLs) each controlling a large proportion of phenotypic variation. In contrast, if the genetic architecture underlying the traits of interest is complex, genomic selection should be preferable [7, 10].

The genetic architecture of quality traits in wheat has been explored in a number of quantitative genetic studies [11–18]. Several QTLs and genes exhibiting large effects have been reported, including *Glu-1* and *Glu-3* [19–22] affecting gluten composition, *Pina-D1* and *Pinb-D1* [23–26] affecting kernel hardness, as well as *Ppo-A1* and *Ppo-D1* [27–29] and *Psy-A1* and *Psy-B1* [30–32] affecting flour color. However, such large effect QTLs are often already fixed in elite breeding programs, as has been exemplified by Groos et al. [18]. Therefore, genomic selection is potentially more relevant than marker-assisted selection to improve quality traits in wheat. This has been confirmed in a pioneering study based on two bi-parental wheat inbred line populations adapted to U.S. environments [33], in which across nine quality traits examined the accuracy of prediction of genomic selection was on average 30% higher than the accuracy of prediction of marker-assisted selection.

Wheat as a selfing species is so far mainly bred as pure line varieties. Implementing hybrid wheat breeding thus holds the potential to boost yield per area and enhance yield stability [34, 35]. One key challenge in the design of a hybrid breeding program is to efficiently select superior hybrids out of millions of potential single cross-combinations [36]. The potential of applying line per se performance or general combining ability effects for predicting wheat hybrid performance have been studied for the traits grain yield, plant height, and heading time as well as susceptibility to frost, lodging, septoria tritici blotch, yellow rust, leaf rust, and powdery mildew [37]. In addition, the limits and prospects of marker-assisted and genomic selection of wheat hybrid performance have been examined for grain yield, plant height, heading date, frost tolerance and resistance to powdery mildew, leaf rust, stripe rust, septoria tritici blotch, and fusarium head blight [38–44]. Nevertheless, options to predict the performance of hybrids for quality traits have not yet been investigated.

Here, we report the results from an approach based on phenotypic data for seven quality traits gathered in field trials conducted in up to six environments and genotypic data generated using a 90k single-nucleotide polymorphism (SNP) array [45] for a large collection of 135 Central European elite winter wheat inbred lines and 1,604 single-cross hybrids derived from them. The objectives were to (1) examine the presence of major effect QTLs for quality traits in the population of 135 parental lines, (2) explore the optimal strategy for predicting hybrid performance for quality traits, and (3) investigate the effects of marker density and the composition and size of the training population on the accuracy of prediction of hybrid performances.

## Materials and Methods

### Plant material and field experiments

The present study was based on 135 elite winter wheat lines adapted to Central Europe and 1,604 F_1_ hybrids derived from them [37, 39]. The hybrids were generated in a factorial crossing scheme with 120 inbred lines serving as female and 15 inbred lines serving as male parents with the aid of chemical hybridization agents. All genotypes were evaluated in up to six environments (S1 Table). The experimental designs were partially replicated alpha designs. All genotypes were randomly split into three adjacent trials linked with 10 common checks and in each trial lines, checks as well as 29% of the hybrids were evaluated in both replications. The sedimentation (SDS) volume according to Zeleny (unit ml) providing a measure for gluten content and swelling properties and thus baking quality of wheat was determined in accordance with International Association for Cereal Science and Technology (ICC) Standards 116/1 and 118 (http://www.icc.or.at/standard_methods). Protein, gluten, and starch content (unit %) as well as hardness (%) were determined by near infrared reflectance (NIR) spectroscopy in accordance to ICC Standard 159 (http://www.icc.or.at/standard_methods). 1000-kernel weight (unit g) and test weight (unit kg hL^-1^) were determined in accordance with the variety registration regulations [46, 47].

### Phenotypic data analyses

After outlier tests [48], we estimated the adjusted entry means for each environment for the following association mapping study based on the following model:

$$y=\mu +X_{G}g+X_{T}t+X_{R}r+X_{B}b+e,$$

where *y* was the vector of phenotypic performance, *μ* was the vector of intercept term, *g* was the vector of genetic effects, *t* was the vector of trial effects, *r* was the vector of replication effects, *b* was the vector of block effects, and *e* was the vector of residuals. *X*_*G*_, *X*_*T*_, *X*_*R*_, and *X*_*B*_ were corresponding design matrices for *g*, *t*, *r*, and *b*. Only *μ* and *g* were treated as fixed effects.

The adjusted means of each genotype across environments were estimated with the following model:

$$y=\mu +X_{G}g+X_{L}l+e,$$

where *y* was the vector of the best linear unbiased estimator (BLUE), *μ* was the vector of intercept term, *g* was the vector of genetic effects, *l* was the vector of environment effects, and *e* was the vector of residuals. *X*_*G*_ and *X*_*L*_ were corresponding design matrices for *g* and *l*, respectively, with only *l* being treated as random effect. For each combination of parental lines, mid-parent value (MP) and relative mid-parent heterosis (MPH) were calculated using hybrid performance (HYB) as follows: MP = (P1 + P2)/2, MPH = [(HYB − MP)/MP] × 100, where P1 and P2 were performance of two parental lines.

The general combining ability (GCA) of parental lines was estimated with the following model:

$$y=\mu +X_{M}gca_{m}+X_{F}gca_{f}+X_{s}sca,$$

where *y* was the vector of BLUEs across environments, *μ* was the vector of intercept term, *gca*_*m*_ was the vector of GCA for male lines, *gca*_*f*_ was the vector of GCA for female lines, *sca* is the vector of special combining ability (SCA) of hybrids. *X*_*M*_, *X*_*F*_ and *X*_*S*_ were corresponding design matrices for *gca*_*m*,_ *gca*_*f*_ and *sca*.

In addition, we estimated the genetic variance components of hybrids and parental lines as well as the variance of genotype × environment interactions by a one-step model:

$$y=\mu +X_{L}l+X_{T}t+X_{R}r+X_{B}b+X_{P}p+X_{PL}z_{pl}+X_{H}h+X_{HL}z_{hl}+e,$$

where *y* was the vector of phenotypic performance, *μ* was the vector of intercept term, *l* was the vector of environment effects, *t* was the vector of trial effects, *r* was the vector of replication effects, *b* was the vector of block effects, *p* was the vector of genetic effects of parental lines, *z*_*pl*_ was the vector of parent-by-environment interaction effects, *h* was the vector of genetic effects of hybrids, *z*_*hl*_ was the vector of hybrid-by-environment interaction effects, and *e* was the vector of residuals. *X*_*L*_, *X*_*T*_, *X*_*R*_, *X*_*B*_, *X*_*P*_, *X*_*PL*_, *X*_*H*_, and *X*_*HL*_ were corresponding design matrices. Except *μ*, all effects were treated as random.

Significance of variance component estimates were tested by model comparison with likelihood ratio tests where the halved P values were used as an approximation [49]. Using the variance components, we estimated the heritability on an entry-mean basis. The phenotypic data analyses were performed using the software ASReml-R 3.0 [50].

### Genotyping

DNA was extracted according to standard procedures from all parental genotypes and fingerprinting was performed with a 90k SNP array based on an Illumina Infinium assay [41]. Hybrid profiles were deduced from the parental fingerprints. All markers that were either monomorphic, had missing values of >5%, heterozygosity of >5% in inbred lines, or had a minor allele frequency of <5% were discarded from analysis. After applying this filtering, 17,372 high-quality SNP markers were retained in the data set [51].

### Genome-wide association mapping and marker-assisted selection

Data from each environment were used in association mapping scans with correction for population stratification with a kinship matrix [39]. The kinship matrices for the inbred lines and hybrids were modeled as described previously [39, 52]. Genome-wide scans for marker-trait associations were conducted to detect main-effect QTLs. The model for association mapping scan [53] is defined as the following:

Y=Xβ+Ss+Zu+e,

where *Y* stands for the adjusted entry means of the 1,739 genotypes within environments, *β* is a vector of environment effects, *s* is a vector of SNP effects, *u* is a vector of polygene background effects, and *e* is a vector of residual effects. *X*, *S*, and *Z* are incidence matrices relating *Y* to *β*, *s*, and *u*. *β* and *s* were treated as fixed effects and *u* as random effect. To check whether the population or family structure was adequately controlled by the model, a QQ-plot was drawn based on the observed P-values and expected P-values of all markers [53].

The Bonferroni-Holm procedure [54] was applied to correct for multiple testing at a significance level (P < 0.05). The association mapping analyses were performed using the software ASReml-R 3.0 [50]. The proportion of the phenotypic variance explained by single QTL (*R*^2^) was estimated using analysis of variance (ANOVA) with QTLs reordered according to the P-values and the proportion of phenotypic variance explained by all the QTLs (*R*^2^_adj_), and effects of detected QTLs were estimated using a standard multiple regression approach [55]. The proportion of explained genotypic variance (*p*_G_) by single QTL and all the QTLs was determined as proportion of explained phenotypic variance standardized by broad-sense heritability (*h*^2^), i.e. *p*_G_ = *R*^2^_adj_/*h*^2^.

### Genomic selection

Based on the adjusted entry means across environments for the 1,739 genotypes included in our study, we applied ridge regression best linear unbiased prediction (RR-BLUP) [56], weighted best linear unbiased prediction (W-BLUP) [40], and Bayes-Cπ [57, 58] considering additive and dominance effects. Details of the implementation of the models have been described in Zhao et al. [39, 40]. Briefly, the general form of the models is defined as the following:

#### RR-BLUP

$$Y=1_{n}\mu +Z_{A}a+Z_{D}d+e,$$

where *Y* stands for the adjusted entry means of the 1,739 genotypes across locations, while *l*_*n*_ is a vector of ones and *n* is the number of genotypes, *μ* refers to the overall mean across all environments, *a* is the additive marker effect, and *d* is the dominance marker effect. *Z*_*A*_ and *Z*_*D*_ are design matrices for the additive and dominance effects of the markers as specified according to the F_∞_ metric of Falconer and Mackay [59], and *e* is the residual. We assumed that additive and dominance marker effects have normal distributions $N\left(0,\;\sigma_{a}^{2}\right)$ and $N\left(0,\;\sigma_{d}^{2}\right)$ with constant variances of additive effects $\sigma_{a}^{2}$ and dominance effects $\sigma_{d}^{2}$. The estimates of *μ*, *a*, and *d*, which are denoted as $\hat{\mu }$, $\hat{a}$, and $\hat{d}$, were obtained from the following mixed-model equation [60]:

$$\left[\begin{array}{c} \hat{\mu }\\ \hat{a}\\ \hat{d} \end{array}\right]={\left[\begin{array}{ccc} 1_{n}^{T}1_{n} & 1_{n}^{T}Z_{A} & 1_{n}^{T}Z_{D}\\ Z_{A}^{T}1_{n} & Z_{A}^{T}Z_{A}+\lambda_{A}I_{m} & Z_{A}^{T}Z_{D}\\ Z_{D}^{T}1_{n} & Z_{D}^{T}Z_{A} & Z_{D}^{T}Z_{D}+\lambda_{D}I_{m} \end{array}\right]}^{-1}\left[\begin{array}{c} 1_{n}^{T}Y\\ Z_{A}^{T}Y\\ Z_{D}^{T}Y \end{array}\right].$$

Here, *I*_*m*_ refers to an identity matrix with dimension of m, where m is the number of markers. The shrinkage parameters *λ*_*A*_ and *λ*_*D*_ were defined as the ratios between the modified variance of residuals ($\frac{\sigma_{e}^{2}}{\text{Nr}.\text{Env}}$) and the marker effects ($\frac{\sigma_{GCA}^{2}}{\text{Nr}.\text{Marker}}$ or $\frac{\sigma_{SCA}^{2}}{\text{Nr}.\text{Marker}}$) [6]. $\sigma_{e}^{2}$, $\sigma_{GCA}^{2}$, and $\sigma_{SCA}^{2}$ represent the variance of residual, GCA effects, and SCA effects. Nr.Env and Nr.Marker represent the number of environments and marker density, respectively.

For a solely additive model, the equation is simplified to:

$$Y=1_{n}\mu +Z_{A}a+e.$$

#### W-BLUP

The model used in W-BLUP is similar to the RR-BLUP model, but we added an additional effect for functional markers, which were defined as the three most significant markers based on association mapping in the training population:

$$Y=1_{n}\mu +Z_{A}a+F_{A}a_{f}+Z_{D}d+F_{D}d_{f}+e,$$

where *a*_*f*_, *d*_*f*_ denote the additive and dominance effects of the functional markers, and *F*_*A*_ and *F*_*D*_ are the design matrices.

#### Bayes-Cπ

Whereas in RR-BLUP it is assumed that all markers contribute to the genetic variance, in Bayes-Cπ only a fraction 1–*π*_*g*_ (*g* denotes either *a* or *d*) of the used markers is considered to contribute to the genetic variance. Based on this assumption, the model for Bayes-Cπ is:

$$Y=1_{n}\mu +Z_{A}\delta_{a}a+Z_{D}\delta_{d}d+e.$$

The additional parameter δ_g_ has a prior distribution:

$$\delta_{g}\;\sim \;\{\begin{array}{c} 0,\;\text{with probability }\pi_{g}\\ 1,\text{ with probability }1-\pi_{g} \end{array}.$$

In Bayes-Cπ, a uniform (0, 1) prior was assumed for *π*_*g*_, resulting in a β-distribution for the full-conditional posterior [58]. For Bayes-Cπ, all above outlined parameters have to be sampled from their full-conditional posterior using a special Markov chain Monte Carlo method called Gibbs sampling.

### Cross-validation of genome-wide association mapping and genomic selection

The prediction accuracy of genotypic values from marker-assisted as well as genomic selection was checked by cross-validation. Due to the factorial mating design of the plant material used in our study, relatedness between estimation and test set was expected to influence prediction accuracy. To account for this effect, we followed the suggestion of Schrag et al. [61] and sampled estimation sets consisting of 10 (out of 15) male and 80 (out of 120) female parental lines as well as 610 (out of potentially 800) hybrids derived from them (S1 Fig). From the remaining hybrids, test sets with three successively decreasing degrees of relatedness to the estimation set were formed. Test set T2 most closely related to the estimation set included only hybrids derived from the same parents as the hybrids that had been evaluated, while the less related test set T1 included hybrids sharing one (either female or male) parent with the hybrids in the estimation set and the least related test set T0 included only hybrids having no parents in common with the estimation set. 100 cross-validation runs were performed for marker-assisted as well as genomic selection and different parental lines, and hybrids derived from them, were selected to compose the estimation set and test sets for each cross-validation run.

For marker-assisted selection, genome-wide association mapping was performed on the sampled estimation set of each cross-validation run. The Bonferroni-Holm procedure [54] was applied to correct for multiple testing. Markers showing trait association at different significance levels (P < 0.10, P < 0.05, P < 0.01, P < 0.001, and P < 0.0001) were selected separately and recorded in order to count the occurrence frequencies of markers. Sizes of effects were estimated for significant marker-trait associations separately in each cross-validation run using a mixed linear model with a random polygenic effect. For genomic selection, marker effects were directly estimated based on the estimation set of each cross-validation run. The obtained marker effects were then used to predict the performance of the hybrids in the T2, T1, and T0 test sets. The accuracy of prediction for each test set was estimated as the Pearson correlation coefficient between the predicted and the observed hybrid performance standardized with the square root of the heritability on an entry-mean basis [5].

### Effect of population size and marker density on prediction accuracy

We applied two further cross-validation strategies to unravel the potential impact of estimation set size and composition on the accuracy of prediction. To validate the impact of the number of parents included, we randomly sampled four different groups of hybrids derived from an increasing number (30, 60, 90, and 120) of female parents and a constant number of 15 male parents to mimic different population sizes. For each size, estimation sets then contained two thirds (20, 40, 60, and 80) of the selected female parents, 10 of the selected male parents, and 100 hybrids derived from them, and the remaining hybrids were split into three test sets according to the relatedness levels (S2 Fig). To examine the influence of number of hybrids on prediction accuracy, we reduced the number of hybrids in estimation sets from 610 as introduced in the first paragraph of last section the cross-validation strategy successively to 500, 300, and 100.

In addition, we randomly sampled k (k from 1 to 173) markers from every 173 markers of the whole marker array in order to follow the accuracy of prediction of genomic selection (RR-BLUP) in dependence on increasing marker density with marker numbers ranging from 100 to 17,300, with intervals of 100. For all outlined cross-validation strategies, 100 runs were performed and the mean of outcomes was calculated.

## Results

### Extensive phenotyping revealed broad genotypic variation and resulted in high heritability

For all seven wheat quality traits examined, we observed a broad variation of phenotypic values across environments (Table 1). Phenotypic values generally followed approximately normal distributions (S3 Fig) consistent with quantitative inheritance. We observed only a low level of average absolute mid-parent heterosis values, with an average of 2% across traits (Table 1). Phenotypic correlation analysis showed that some traits were highly related, e.g., gluten content was significantly (P < 0.001) related with protein content with a correlation coefficient of 0.81 (S4 Fig). For all seven quality traits, the wide genetic variation present resulted in significant genotypic variances (P<0.001) for the parental lines and as well as the hybrids (Table 1). Heritability estimates across all the environments were moderate to high (ranging from 0.63 for starch content to 0.96 for SDS volume) and were much higher than plot-based heritability. Thus, field testing at several environments was required to obtain high quality phenotypic data.

**Table 1:** First and second degree statistics for 135 inbred lines and 1,604 hybrids derived from them for quality traits gluten content(%), kernel hardness (%), protein content (%), SDS value (ml), starch content (%), test weight (kg/hL), and 1000-kernel weight (g) determined in up to six environments.

	Gluten content	Kernel hardness	Protein content	SDS value	Starch content	Test weight	1000-kernel weight
Environments	2	3	6	5	2	3	4
σ²_environment_	3.75***	206.21***	1.26***	120.23***	1.46*	4.53*	1.36
σ²_trial_	0.00	0.16	0.01*	0.98*	0.07*	0.25*	0.62*
σ²_replication_	0.02***	1.46***	0.01***	1.24***	0.01**	0.07***	0.34***
σ²_block_	0.18***	2.69***	0.08***	2.22***	0.06***	1.23***	0.49***
Lines							
Mean	27.6	46.7	12.4	44.1	68.1	75.8	45.3
Range	24.6–33.2	20.8–61.5	11.6–14.0	28.2–58.9	66.4–69.6	66.6–79.7	38.9–54.1
σ²_Lines_	1.42***	38.80***	0.18***	47.99***	0.44***	3.99***	8.18***
σ²_Lines×Environment_	0.56***	26.50***	0.12***	6.88***	0.20***	0.38***	2.98***
*h*²_Lines_	0.78	0.78	0.87	0.96	0.72	0.91	0.89
Hybrids							
Mean	27.2**	50.1***	12.0***	43.1	68.6***	77.3***	48.4***
Range	24.5–30.5	30.7–65.6	11.1–13.2	26.7–55.0	65.7–70.2	68.0–80.9	42.3–56.4
σ²_Hybrids_	0.61***	13.36***	0.08***	22.79***	0.15***	1.10***	3.53***
σ²_Hybrids×Environment_	0.04	12.53***	0.02***	1.59***	0.01	0.58***	1.35***
σ²_error_	0.47	12.36	0.09	6.99	0.28	1.61	2.03
MPH (%)	-0.89	3.53	-1.97	-2.36	0.51	1.43	5.85
*h*²_Hybrids_	0.76	0.70	0.84	0.94	0.63	0.64	0.83
*h*²_Lines_^#^	0.58	0.50	0.47	0.78	0.48	0.67	0.62
*h*²_Hybrids_^#^	0.55	0.42	0.42	0.73	0.41	0.33	0.51

*, **, and *** indicate *P* < 0.05, *P* < 0.01 and *P* < 0.001 levels of probability, respectively, for means of hybrids being significantly different from means of lines or σ² values being significantly different from zero, respectively. MPH indicates average mid parent heterosis in hybrids.

^#^ represents heritability on a plot basis.

### Association mapping indicated putative QTLs for all seven quality traits

The quantile-quantile plot revealed absence of an excess of QTL (S5 Fig), indicating that population stratification had been sufficiently controlled. QTLs with additive effects were detected for all seven quality traits (S2 Table). Eight markers with additive effects were shared by gluten content and protein content, implying a close correlation between these two traits. No marker with dominance effect was detected for gluten content, kernel hardness, and starch content. Only 4% of the detected markers showed significant interaction effects with the environments. Markers explaining more than 10% of the genotypic variation were observed for the five quality traits gluten content, kernel hardness, protein content, SDS volume, and 1000-kernel weight. The total proportion of genotypic variance explained by all putative QTL exceeded 30% for each of these five traits, while it was lower for starch content and test weight.

For all quality traits except starch content and test weight, the detected markers with significant (P < 0.05 applying Bonferroni-Holm correction) additive effects were distributed over different chromosomes (S2 Table). None of the detected marker-trait associations has been reported in available QTL studies, but most of them were located in chromosomal regions that were previously described to be related to quality traits (S2 and S3 Tables). To check whether the significant markers were located in transcribed regions of the wheat genome, local blast was performed to align marker sequences with wheat cDNA sequences. In total, 35 markers were successfully aligned to cDNA sequences with identities >99% (S4 Table). These cDNA sequences could identify putative candidate genes related to quality traits, although further efforts are required to confirm this assumption.

**Fig 1:**
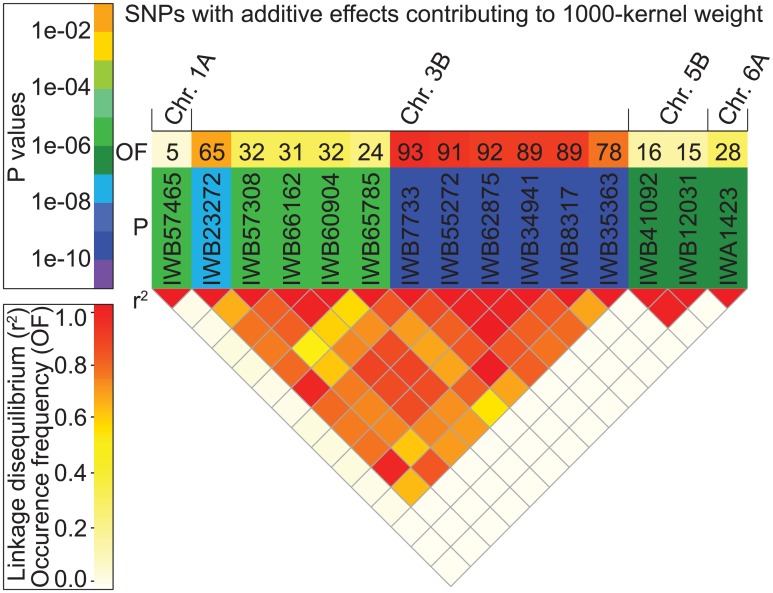
SNPs with additive effects contributing to 1000-kernel weight. Heat-plots of (OF, top row) frequencies with which SNP markers were significantly associated with 1000-kernel weight in 100 cross-validation runs, (P, middle row) P-values of respective SNP markers that contributed significantly to the additive genetic variation of 1000-kernel weight, and (r², lower triangular section) linkage disequilibrium measured as squared Pearson’s correlation coefficients among SNP markers.

Most of the markers significantly associated to 1000-kernel weight were centered on chromosome 3B (Fig 1). Ten of the 15 detected markers for 1000-kernel weight were positioned in a window of 7 cM (S2 Table) and strong linkage disequilibrium (*r*^*2*^) was detected among these markers (Fig 1). The allele effect of the most significant marker underlying 1000-kernel weight amounted to -6.5 g (S2 Table), which is substantial when considering the given range of BLUEs with a minimum of 42.3 g and a maximum of 56.4 g.

### Non-cross validated accuracy of marker-assisted selection is severely overestimated

**Fig 2:**
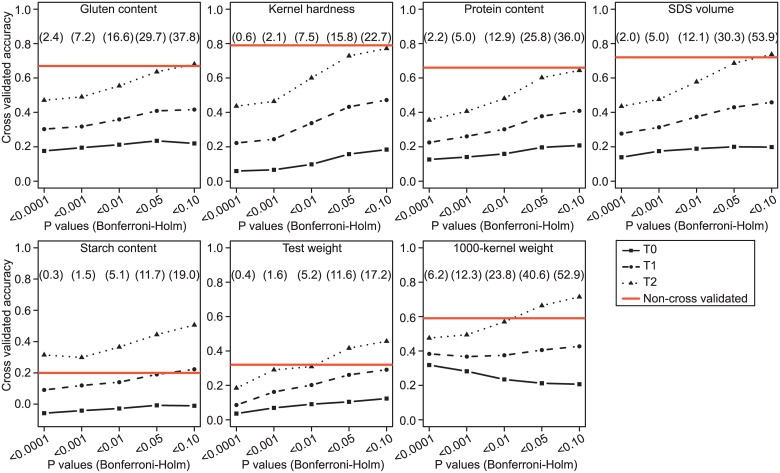
Cross-validated accuracies of prediction in marker-assisted selection for seven wheat quality traits. Varying degrees of relatedness between the estimation and test sets, with triangles indicating a T2 scenario with closest, round discs a T1 scenario with intermediate, and squares a T0 scenario with closest relatedness, and levels of significance, with P-values of 0.001, 0.001, 0.01, 0.05, 0.1, and applying Bonferroni-Holm correction for multiple testing, were used in genome-wide scans for marker-trait associations. Significant markers were then used to predict the performance of the individuals included in test sets. Numbers in brackets indicate the average number of significant marker-trait associations found based on 100 cross-validation runs. Red lines show corresponding non-cross-validated accuracies of prediction accuracies obtained based on the full data set.

Cross-validated accuracies of prediction through marker-assisted selection increased with relaxing significance threshold for all traits and cross-validation scenarios except for the T0 scenario and 1000-kernel weight (Fig 2). Moreover, the accuracies of prediction decreased gradually with increasing genetic distance between estimation and test sets. For the T0 scenario, in which the relatedness was lowest, comparison of accuracies without and with cross-validation revealed a substantial overestimation of accuracies of prediction by marker-assisted selection for all seven quality traits analyzed (Table 2; S2 Table). In contrast, the overestimation of the accuracy of prediction was small in the case of the most related T2 scenario.

**Table 2:** Cross-validated accuracies of prediction of marker-assisted selection, genomic selection, mid-parent prediction and prediction based on general-combining ability effects.

Trait	MAS^a^	MAS-CV^b^			RR-BLUP			Bayes-Cπ			W-BLUP			MP^c^	GCA^d^
		T0	T1	T2	T0	T1	T2	T0	T1	T2	T0	T1	T2	T2	T2
Gluten content	0.67	0.23	0.41	0.64	0.37	0.69	0.95	0.36	0.69	0.95	0.36	0.68	0.95	0.88	0.94
Kernel hardness	0.79	0.16	0.43	0.73	0.34	0.77	1.00^e^	0.35	0.78	1.00^d^	0.29	0.74	1.00^e^	0.97	1.00^d^
Protein content	0.66	0.19	0.38	0.50	0.36	0.69	0.96	0.35	0.69	0.96	0.34	0.68	0.96	0.89	0.95
SDS value	0.72	0.20	0.43	0.69	0.48	0.79	0.99	0.48	0.79	0.99	0.47	0.78	0.99	0.97	0.98
Starch content	0.20	-0.01	0.19	0.44	0.27	0.64	0.89	0.28	0.65	0.90	0.20	0.60	0.89	0.80	0.88
Test weight	0.32	0.11	0.26	0.42	0.41	0.67	0.89	0.44	0.69	0.89	0.34	0.63	0.88	0.78	0.87
1000-kernel weight	0.59	0.21	0.41	0.66	0.39	0.74	0.98	0.38	0.74	0.98	0.40	0.73	0.98	0.92	0.97

^a^ accuracy of prediction based on marker-assisted selection without cross-validation at significance level *p-value* < 0.05 (Bonferroni-Holm correction).

^b^ accuracy of prediction based on marker-assisted selection with cross-validation at significance level *p-value* < 0.05 (Bonferroni-Holm correction).

^c^ MP represents cross-validated mid-parent prediction.

^d^ GCA represents cross-validated prediction based on general-combining ability effects.

^e^ prediction accuracies above 1 were set as 1.

We also counted the occurrence frequency of each particular QTL within 100 cross-validation runs (Fig 1). For all seven quality traits except 1000-kernel weight, unstable putative QTLs detected in less than 50% of the runs or moderately stable putative QTLs detected in less than 80% of the runs were detected. In contrast, five stable marker-trait associations were detected for 1000-kernel weight with a high occurrence frequency of nearly 90 out of 100 cross-validation runs.

### Genomic selection outperformed marker-assisted selection in terms of accuracies of prediction

The cross-validated accuracies of prediction of genomic selection by RR-BLUP were in all cases substantially higher than that of marker-assisted selection (Table 2). The trends of accuracies obtained by RR-BLUP in T2, T1, and T0 scenarios were in accordance with the observations made for marker-assisted selection. For the T0 scenario, in which the test set was least related to the estimation set, we observed the lowest accuracies of prediction. In contrast, we observed highest accuracies of predictions for the T2 scenario with closest relatedness to the estimation set.

Our cross-validation study revealed 1000-kernel weight as the only trait for which stable putative QTLs were obtained. Implementation of Bayes-Cπ and W-BLUP models was expected to improve the accuracy of prediction. Nevertheless, we observed no increase in the accuracy of prediction accuracy for Bayes-Cπ and only a slight increase of 2.6% in the case of the T0 scenario for W-BLUP compared to RR-BLUP (Table 2).

In addition, we observed in 81% of the cross-validation runs higher accuracies of prediction for the additive plus dominance model compared with the pure additive model based on the same training population of hybrids. Nevertheless, the benefits in accuracies were only marginal with an average of 0.27%.

### Effects of marker density and estimation population size on the accuracy of prediction

**Fig 3:**
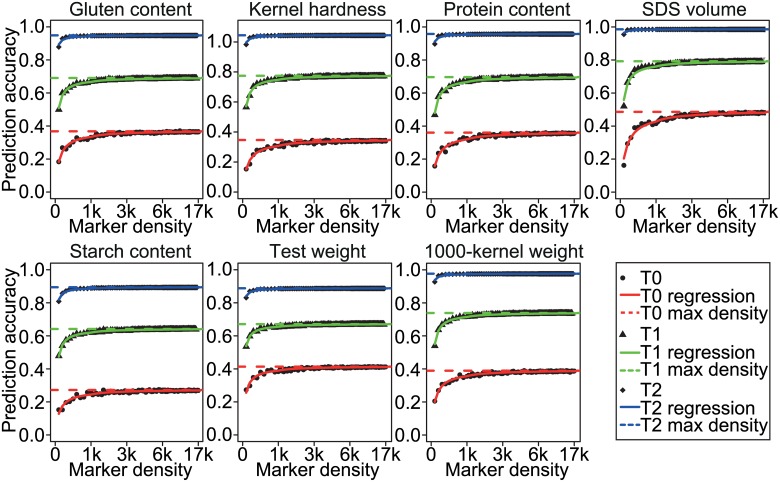
Cross-validated accuracies of prediction of genomic selection using RR-BLUP for seven quality traits in dependence on marker density (0.1-17k) for three cross-validation scenarios. The dashed lines indicate accuracies of prediction observed with highest marker density as reference, blue, green, and red refer to T2, T1, and T0 scenarios with closest, intermediate, and lowest relatedness between estimation and test sets, respectively.

We studied the effect of the marker density on the prediction accuracy based on an equidistant sampling of subsets of SNPs. The prediction accuracies enhanced with increasing marker density for the T2, T1, and T1 scenarios (Fig 3). The increase plateaued, however, after ~3k for the T0 scenario, after ~2k for the T1 scenario, and 0.5k for the T2 scenario.

**Fig 4:**
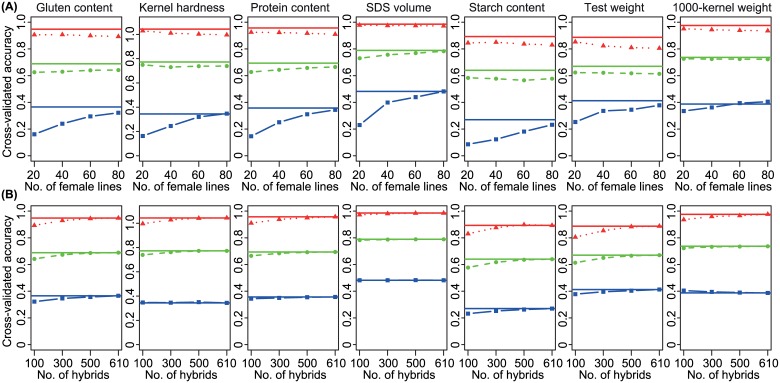
Cross-validated accuracies of prediction of genomic selection using RR-BLUP for seven quality in dependence on the size and composition of estimation sets as well as the relatedness between estimation and test sets. Estimation sets consisted of (A, top row) 10 male lines, a varying number of female lines, and 100 hybrids derived from them or (B, lower row) 10 male parents, 80 females, and varying numbers of hybrids derived from them. Triangles in red, round discs in green, and squares in blue represent T2, T1, and T0 scenarios with closest, intermediate, and lowest relatedness between estimation and test sets, respectively. Solid red, green, and blue lines indicate accuracies for of prediction for T2, T1, and T0 scenarios with estimation sets consisting of 10 male parents, 80 female parents, and 610 hybrids as reference.

We observed a substantial increase in the accuracy of prediction for the T0 scenario from 0.20 for in total 30 parental lines to 0.36 for in total 90 parental lines in the estimation set while keeping the number of hybrids (100) and male parents (10) constant (Fig 4A). In contrast, the accuracies of prediction increased only marginally for the T1 and T2 scenarios when increasing the number of parental lines included. Increasing the number of hybrids but keeping the number of parental lines constantled for T0, T1, and T2 scenarios to only a slight increase in accuracies of prediction, with a maximum increase of 12% for starch content when increasing the number of hybrids from 100 to 610 (Fig 4B).

## Discussion

### Absence of robust QTLs hampers marker-assisted selection of quality traits in elite wheat except for 1000-kernel weight

Marker-assisted selection is beneficial compared to phenotypic selection in particular for (1) traits that are controlled by major effect QTLs [62, 63] and (2) traits that are expensive to phenotype [64, 65]. We observed low accuracies of prediction in the case of low relatedness between estimation and test sets as with the T0 cross-validation scenario (Fig 2) and a prevalence of unstable marker-trait associations (S2 Table), which clearly suggests absence of large effect QTL for all traits studied except 1000-kernel weight. This is not surprising, as large effect QTL are likely to be fixed in elite germplasm for important quality traits as has been reported for instance for a RIL population of French elite wheat lines [18]. Consequently, our findings suggest low prospects of marker-assisted selection for gluten content, kernel hardness, protein content, SDS volume, starch content, and test weight in Central European wheat breeding.

For 1000-kernel weight, a robust putative QTL explaining 18% of genotypic variation was detected on chromosome 3B. The chromosomal region corresponds to a previously reported QTL for 1000-kernel weight [66]. Accordingly, applying the most stringent significance threshold (P < 0.0001), we observed for 1000-kernel weight the highest accuracy of prediction for the T0 scenario (0.32), and the smallest difference between T0 and T2 scenarios (0.16) among all analyzed traits (Fig 2). These findings in combination with the high occurrence frequency of respective marker-trait associations (Fig 1) clearly suggest presence of a reliable, large effect QTL for 1000-kernel weight which provides a very interesting target for further fine-mapping and map-based cloning activities. Interestingly, the large effect QTL has not yet been fixed in the selection process, which may be explained by a low contribution of 1000-kernel weight to grain yield.

### Genomic selection for quality traits based on RR-BLUP is only for the T0 and T1 scenarios an attractive alternative/complementation to phenotypic selection

Several studies performed on wheat inbred line populations reported moderate to high accuracies of prediction by genomic selection for a wide array of traits in wheat [33, 38, 67–69]. In accordance with these findings, in our study using hybrid wheat, for the T1 scenario involving intermediate relatedness and the T2 scenario involving high relatedness between estimation and test sets, we observed moderate to high accuracies of prediction, which points to the potential of genomic selection for improving quality traits. Heffner et al. [33] had used two segregating populations of wheat lines adapted to the U.S., which were genotyped with 399 to 574 molecular markers and phenotyped for nine quality traits. In this setup, genomic selection substantially outperformed marker-assisted selection for line *per se* performance with an average increase of 30%. Our results for genomic selection in hybrid prediction (Table 2) are in line with this observation and are consistent with the presence of multiple QTLs each exhibiting only small effects [6]. Consequently, genomic selection is preferred to marker-assisted selection for improving the quality of wheat hybrids.

Hybrid breeding facilitates to exploit dominance effects in contrast to line breeding [59], which is reflected by a 8.1% smaller prediction accuracy by training the prediction model purely based on parental lines as compared to using hybrid data (data not shown). Therefore, Technow et al. [70] recommended to predict hybrid performance by exploiting both additive and dominance effects. We observed only marginal benefits by using additive plus dominance model compared with the pure additive model. The only marginal advantages were not due to a tight correlation between the additive and dominance relationship matrices, which actually was low (r = 0.21), but rather can be explained by the low ratio of variance of specific versus general combining ability effects for quality traits [34]. In addition, dominance effects are intra-locus interaction effects and are, hence, more difficult to estimate than additive effect, which are main effects [38, 39, 70].

Hybrid prediction can also be based on mid-parent performance or general combining ability effects. These data, however, are only available under certain conditions: mid-parent prediction requires that phenotypic data are available for both parental lines of the hybrids and GCA prediction requires that both parents of evaluated hybrids were involved in the estimation hybrid set (for review, see Schrag et al. [71]). The tiny differences that were observed when comparing MP- and GCA-based accuracies of prediction with that based on RR-BLUP clearly points to the overwhelming importance of additive versus dominance effects for quality traits. In particular, GCA-based prediction could be hardly outperformed by applying genomic selection (Table 2). Consequently, for the T2 scenario, genomic selection is in terms of accuracy of prediction not an attractive alternative to phenotypic selection.

For the T0 and T1 scenarios, MP- and GCA-based prediction is not applicable, while genomic selection is a potential strategy to predict hybrid performance. Longin et al. [72] suggested based on a simulation study that hybrid breeding applying exclusively genomic selection can only be recommended if prediction accuracies exceed 0.5, in which case purely genomic selection strategy showed maximum annual gain compared to the other breeding strategies. Our results revealed that a prediction accuracy of 0.5 is only reached in the T1 but not in the T0 scenario for RR-BLUP (Table 2). Consequently, genomic selection is only for the T1 scenario the method of choice and must be combined with phenotypic selection for wheat quality for the T0 scenario.

### Genomic selection approaches designed to more properly model a mix between large and small effect QTLs did not boost accuracy of prediction of quality traits

Previous simulation studies have shown that equal shrinkage of marker effects as applied in RR-BLUP can result in too strict shrinkage for QTL exhibiting large effects [6, 73]. Bayesian models allow specific shrinkage of every marker [74], and, thus, are expected to outperform RR-BLUP if large effect QTLs for the relevant trait are present. Among the seven quality traits examined in our study, 1000-kernel weight is the only trait for which presence of a QTL exhibiting a large effect is indicated (Figs 1 and 2). Despite the presence of large effect QTL, however, Bayes-Cπ did not outperform RR-BLUP. This can be explained by still too strict shrinkage [40] or by high marker density resulting in a bulk of markers in linkage disequilibrium to the major QTL [7, 75]. To distinguish between both reasons, we adopted an further alternative approach W-BLUP, which was in a previous study successfully used to enhance accuracy of prediction for heading time and plant height [40]. In contrast to the previous results, we observed in our current study no significant increase in accuracy of prediction when applying W-BLUP compared with RR-BLUP (Table 2). Rather, closer examination of the QTL region on chromosome 3B revealed that the large-effect QTL is actually captured through a number of closely linked SNPs (Fig 1), explaining the lack of an increase in prediction accuracies switching from RR-BLUP to Bayes-Cπ or W-BLUP.

### Increasing the number of parental lines in the estimation set boosts prediction accuracies in the T0 scenario

Meuwissen [76] reported that the accuracies of prediction of the performance of distantly related individuals were restricted by both marker density and phenotypic records of the estimation set. We performed an equidistant sampling of subsets of SNPs and found that the accuracy of prediction plateaued after ~3k (of approx. 17k available) for the T0 scenario with low relatedness between estimation and test set (Fig 3), indicating that marker density is not a limiting factor in our study. To take advantage of an increase in the marker density, large estimation sets are required [76]. Two alternative strategies, i.e. increasing the number of parental lines or hybrids, are possible to enlarge the estimation set used for hybrid prediction. We observed increasing accuracies of prediction for both strategies in the T0 scenario (Fig 4). The increase in accuracy was much stronger pronounced when the number of parental lines was increased (Fig 4A) compared to enhancing the number of hybrids included, while keeping the number of parents constant (Fig 4B). This is not surprising and can be explained by the key role of the effective population size in driving accuracies of prediction [76]. We had sampled a set of diverse 135 parental wheat lines for out wheat hybrid approach [37] and thus by increasing the number of parents, also the effective population size was likely to increase. In contrast, keeping the number of parents constant while increasing the number of hybrids did not result in an enhanced effective population sizes. Consequently, an economic and efficient way to improve accuracy of prediction of the hybrid performances in the T0 scenario should use a large number of genetically diverse parents, which are evaluated in only a reduced number of hybrid combinations.

## Conclusion

For all seven quality traits scrutinized, association mapping detected QTLs, but cross-validation revealed a large overestimation of prediction accuracies of marker-assisted selection. Genomic selection generally showed improved prediction accuracies compared to marker-assisted selection and is the method of choice especially when predicting the performance of hybrids of T0 and T1 population. Prediction accuracies can be maximized when enlarging rather the number of parents in the training population than increasing the number of hybrid combinations per parent.

## Supporting Information

S1 Fig Scheme for the allocation of hybrids in cross-validation to estimation sets as well as T2, T1, and T0 test sets with successively decreasing degree of relatedness to estimation sets. Estimation sets comprised random selections of 80 out of 120 female (F) and 10 out of 15 male (M) parental inbred lines as well as 610 hybrids derived from them. Test sets included only hybrids not assigned to the respective estimation set that had either both parents (T2), one parent (T1) or no parent (T0) in common with the hybrids.
(EPS)

S2 Fig Scheme for subpopulation sampling and allocation of hybrids within subpopulation to estimation sets. (EPS)

S3 Fig Distributions of phenotypic values for seven quality traits based on an evaluation of 135 parental inbred lines and 1,604 hybrids in up to six environments. Arrows indicate means of values.
(EPS)

S4 Fig Correlation of BLUEs across environments. The upper right triangular section shows correlations among quality trait values; the lower left triangular sections plot maps for each pair of quality trait values; while the diagonal shows the histogram and density lines of quality trait values. *, **, and *** indicate *P* < 0.05, *P* < 0.01 and *P* < 0.001 levels of probability for phenotypic performance of different traits being significantly correlated.
(EPS)

S5 Fig Quantile-quantile plots for association mapping based on the combined parental lines and hybrids. Four different biometrical approaches were performed: (1) without correcting for population structure, (2) with correcting for population structure with a heterotic effect, (3) with correcting for population structure with a kinship matrix, and (4) with correcting for population structure with a kinship matrix and a heterotic effect.
(EPS)

S1 Table Summary of environments and genotypes involved in field experiments for each quality trait. (DOCX)

S2 Table Significant (*P* < 0.05 and Bonferroni-Holm correction) marker-trait associations, and the proportion of genotypic variance (*p*_*G*_) explained by them, that were detected in a genome-wide association mapping approach for seven quality traits in a Central European winter wheat population based on a 90k SNP array. (DOCX)

S3 Table Putative QTLs for wheat quality traits reported in previous studies. (DOCX)

S4 Table Detailed information on markers with significant trait-association that could be successfully aligned to wheat coding sequence data. (XLSX)
