# Supplementary material for: Predicting Hybrid Performances for Quality Traits through Genomic-Assisted Approaches in Central European Wheat
Source: PLoS One. 2016 Jul 6;11(7):e0158635. doi: 10.1371/journal.pone.0158635 (PMC4934823; doi:10.1371/journal.pone.0158635)
Supplement: S1 Table — (DOCX) [file pone.0158635.s006.docx]

**S1 Table.** **Summary of environments and genotypes involved in field experiments for each quality trait.**

| Environment | Gluten content | Kernel hardness | Protein content | SDS values | Starch content | Test weight | 1000-kernel weight |
| --- | --- | --- | --- | --- | --- | --- | --- |
| Böhnshausen (Boh12) |  | 94 lines and 1568 hybrids | 94 lines and 1568 hybrids | 94 lines and 1568 hybrids |  | 94 lines and 1568 hybrids | 94 lines and 1568 hybrids |
| Hadmersleben (Had12) | 135 lines and 1604 hybrids | 135 lines and 1604 hybrids | 135 lines and 1604 hybrids | 135 lines and 1604 hybrids | 135 lines and 1604 hybrids | 135 lines and 1604 hybrids |  |
| Hohenheim (Hoh12) |  |  | 135 lines and 1604 hybrids | 135 lines and 1604 hybrids |  |  | 135 lines and 1604 hybrids |
| Adenstedt (Ade13) |  |  | 135 lines and 1604 hybrids |  |  |  |  |
| Hadmersleben (Had13) | 135 lines and 1604 hybrids | 135 lines and 1604 hybrids | 135 lines and 1604 hybrids | 135 lines and 1604 hybrids | 135 lines and 1604 hybrids | 135 lines and 1604 hybrids | 135 lines and 1604 hybrids |
| Seligenstadt (Sel13) |  |  | 135 lines and 1604 hybrids | 135 lines and 1604 hybrids |  |  | 135 lines and 1604 hybrids |
