# Supplementary material for: Predicting Hybrid Performances for Quality Traits through Genomic-Assisted Approaches in Central European Wheat
Source: PLoS One. 2016 Jul 6;11(7):e0158635. doi: 10.1371/journal.pone.0158635 (PMC4934823; doi:10.1371/journal.pone.0158635)
Supplement: S2 Table — (DOCX) [file pone.0158635.s007.docx]

**S2 Table.** **Significant (*P* < 0.05 and Bonferroni-Holm correction) marker-trait associations, and the proportion of genotypic variance (*p_G_*) explained by them, that were detected in a genome-wide association mapping approach for seven quality traits in a Central European winter wheat population based on a 90k SNP array.**

| 90k SNP array | Marker name | P Value^a^ | *p_G_*(%) | Allele effect | Frequency^b^ | Chromosome position (cM) |
| --- | --- | --- | --- | --- | --- | --- |
| Gluten content | | | | | | |
| Additive | IWB65782 | 1.01E-09 | 22.83 | -0.47 | 64 | 3A 269.13 |
|  | IWA6877 | 1.03E-09 | 7.11 | 0.58 | 69 | 3A 274.44 |
|  | IWB2330 | 1.43E-09 | 1.26 | -1.46 | 65 | 3A 269.13 |
|  | IWB9225 | 4.74E-08 | 0.01 | 0.93 | 43 | 3A* |
|  | IWA2618 | 7.63E-08 | 2.34 | -3.10 | 51 | 3A 280.36 |
|  | IWB4728 | 1.62E-07 | 0.79 | 0.68 | 32 | 3A 288.43 |
|  | IWB58235 | 2.98E-07 | 1.16 | -0.64 | 22 | 3A 218.69 |
|  | IWB10336 | 7.67E-07 | 0.49 | 0.10 | 30 | 3A 280.36 |
|  | IWB33701 | 7.96E-07 | 1.42 | 0.14 | 26 | Not mapped |
|  | IWB35281 | 8.75E-07 | 1.96 | 0.54 | 25 | 3A 274.44 |
|  | IWB30893 | 9.96E-07 | 3.20 | -0.21 | 14 | 4B 178.41 |
|  | IWB7165 | 1.15E-06 | 0.20 | 0.83 | 18 | 3A 218.69 |
|  | IWA2978 | 1.25E-06 | 0.51 | 0.06 | 0 | 3B 323.92 |
|  | IWA8526 | 1.45E-06 | 0.01 | 0.21 | 23 | 3A 274.44 |
|  | IWA1998 | 1.51E-06 | 0.09 | 0.02 | 21 | 3A 284.21 |
|  | IWB13889 | 1.78E-06 | 2.07 | 2.95 | 29 | 3A 280.36 |
|  | IWA4146 | 2.15E-06 | 0.03 | -0.29 | 1 | 3B 323.92 |
|  | Total^c^ |  | 44.67 |  |  |  |
| Kernel hardness | | | | | | |
| Additive | IWB70794 | 6.34E-08 | 14.34 | -2.07 | 32 | 4B 46.43 |
|  | IWB54831 | 7.51E-08 | 5.17 | -1.14 | 40 | 2B* |
|  | IWB74228 | 5.20E-07 | 0.76 | -0.65 | 14 | 4B 46.43 |
|  | IWB74123 | 5.66E-07 | 12.60 | -2.40 | 44 | 7A 125.94 |
|  | IWA2545 | 8.77E-07 | 4.51 | 2.09 | 21 | 7D 207.41 |
|  | IWB57894 | 9.27E-07 | 3.19 | 2.55 | 18 | 1A 439.42 |
|  | IWB10582 | 9.90E-07 | 2.96 | -0.52 | 15 | 1B 415.34 |
|  | IWB41295 | 1.20E-06 | 3.07 | -1.42 | 13 | 2D 133.17 |
|  | IWB10246 | 1.46E-06 | 7.94 | -1.48 | 20 | 3B* |
|  | IWB60997 | 1.62E-06 | 4.21 | -0.07 | 20 | 7A 624.47 |
|  | IWB10118 | 1.77E-06 | 0.16 | 4.40 | 20 | 7A 624.47 |
|  | IWB7857 | 1.83E-06 | 0.43 | -2.92 | 13 | Not mapped |
|  | IWB71178 | 2.04E-06 | 0.09 | -1.45 | 18 | 7A 624.47 |
|  | IWB5573 | 2.06E-06 | 1.39 | 1.93 | 16 | 6A 145.02 |
|  | IWB31739 | 2.15E-06 | 0.94 | 1.33 | 13 | Not mapped |
|  | IWB5905 | 2.56E-06 | 0.13 | -0.20 | 15 | Not mapped |
|  | IWB10212 | 2.58E-06 | 0.76 | 1.51 | 12 | 7A 624.47 |
|  | IWB72348 | 2.84E-06 | 0.04 | 0.91 | 11 | 4B 46.43 |
|  | Total^c^ |  | 61.87 |  |  |  |
| Protein content | | | | | | |
| Additive | IWB2330 | 1.66E-09 | 17.29 | -0.61 | 49 | 3A 269.13 |
|  | IWA6877 | 2.66E-09 | 10.15 | 0.23 | 70 | 3A 274.44 |
|  | IWB65782 | 6.22E-09 | 0.58 | -0.43 | 41 | 3A 269.13 |
|  | IWB14791 | 1.87E-07 | 3.74 | -0.09 | 22 | 1B 374.33 |
|  | IWB74975 | 2.45E-07 | 1.87 | 0.17 | 20 | 3A 47.94 |
|  | IWB74547 | 3.18E-07 | 0.00 | 0.02 | 21 | 1B* |
|  | IWB35281 | 5.83E-07 | 0.52 | 0.00 | 28 | 3A 274.44 |
|  | IWA2978 | 7.27E-07 | 0.33 | -0.10 | 0 | 3B 323.92 |
|  | IWB9225 | 7.96E-07 | 0.00 | -0.05 | 19 | 3A* |
|  | IWB7003 | 8.10E-07 | 2.10 | -0.04 | 9 | 3A 82.86 |
|  | IWA8526 | 8.40E-07 | 0.18 | -0.08 | 20 | 3A 274.44 |
|  | IWB61173 | 1.40E-06 | 2.13 | -0.06 | 6 | 5B 349.64 |
|  | IWB56482 | 1.57E-06 | 0.02 | -0.11 | 2 | 3A 391.81 |
|  | IWB10638 | 1.74E-06 | 0.05 | -0.08 | 7 | 3A 82.86 |
|  | IWB65832 | 1.77E-06 | 3.12 | 0.05 | 9 | 1B 224.75 |
|  | IWB27784 | 1.91E-06 | 1.35 | 0.06 | 14 | 1B 315.61 |
|  | IWA4146 | 2.20E-06 | 0.04 | 0.10 | 0 | 3B 323.92 |
|  | IWB72533 | 2.84E-06 | 0.74 | -0.13 | 12 | 1B 227.17 |
|  | Total^c^ |  | 43.42 |  |  |  |
| Dominance | IWB65693 | 7.74E-12 | 0.15 | 0.20 | 26 | 5A 709.71 |
|  | IWB12585 | 1.69E-11 | 0.08 | -0.24 | 24 | 4B 335.17 |
|  | IWB6768 | 2.43E-11 | 0.02 | -0.03 | 24 | 5A 709.71 |
|  | IWB65779 | 4.22E-11 | 0.27 | 0.14 | 25 | 5A 709.71 |
|  | IWA6447 | 1.52E-10 | 0.00 | 0.21 | 12 | 5B 126.02 |
|  | IWB41946 | 2.78E-10 | 0.11 | 0.07 | 11 | 5B* |
|  | IWB73859 | 5.95E-10 | 0.45 | -0.30 | 10 | 5B 130.15 |
|  | IWB9800 | 7.11E-10 | 0.12 | -0.03 | 16 | 5A 709.71 |
|  | IWB10387 | 8.75E-09 | 0.04 | -0.04 | 17 | 5A 709.71 |
|  | IWB24813 | 1.03E-08 | 0.02 | -0.03 | 15 | 5A 699.72 |
|  | IWB74923 | 1.08E-08 | 0.06 | 0.17 | 16 | 5A 699.72 |
|  | IWB24570 | 4.10E-08 | 0.00 | -0.03 | 13 | 5A 699.72 |
|  | IWB74922 | 8.50E-08 | 0.12 | -0.13 | 12 | 5A 699.72 |
|  | IWB235 | 9.10E-08 | 0.05 | 0.05 | 11 | 5A 742.53 |
|  | IWB38693 | 9.10E-07 | 0.46 | -0.42 | 4 | 1B 285.05 |
|  | IWB33010 | 1.05E-06 | 0.05 | -0.15 | 4 | 5A 742.53 |
|  | IWB5731 | 1.11E-06 | 0.01 | 0.08 | 9 | 5A 737.3 |
|  | IWA3189 | 2.33E-06 | 0.00 | 0.42 | 2 | 1B 278.7 |
|  | Total^c^ |  | 0.82 |  |  |  |
| SDS value | | | | | | |
| Additive | IWB47978 | 8.93E-09 | 23.24 | 2.87 | 78 | 1B 49.56 |
|  | IWB6944 | 3.44E-08 | 2.45 | 13.64 | 45 | 1A 264.58 |
|  | IWB11087 | 1.10E-07 | 0.85 | 0.62 | 40 | 1A 264.58 |
|  | IWB6974 | 1.18E-07 | 1.81 | -10.44 | 37 | 1A 264.58 |
|  | IWB24303.1 | 2.47E-07 | 1.19 | 10.13 | 33 | 1B 142.58 |
|  | IWB35107 | 2.83E-07 | 0.72 | 7.90 | 28 | 1B* |
|  | IWB10582 | 2.99E-07 | 5.41 | -3.03 | 29 | 1B 415.34 |
|  | IWA605 | 3.34E-07 | 0.07 | 1.79 | 36 | 1A 260.38 |
|  | IWB44606 | 1.15E-06 | 1.98 | 1.96 | 9 | 1B 415.34 |
|  | IWB74583 | 1.16E-06 | 0.02 | 1.18 | 25 | 1B 142.58 |
|  | IWB73709 | 1.55E-06 | 8.84 | -1.90 | 19 | 5A 445.69 |
|  | IWB34802 | 1.60E-06 | 0.10 | 0.33 | 33 | 1B 71.09 |
|  | IWB31739 | 1.60E-06 | 0.00 | 0.46 | 14 | Not mapped |
|  | IWB35518 | 2.49E-06 | 0.00 | -0.60 | 15 | 1B* |
|  | IWB25528 | 2.54E-06 | 0.97 | 1.47 | 8 | 7B 10.46 |
|  | IWB63759 | 2.81E-06 | 2.69 | 1.57 | 4 | 2B* |
|  | Total^c^ |  | 49.84 |  |  |  |
| Dominance | IWB24813 | 4.36E-12 | 0.14 | 2.75 | 23 | 5A 699.72 |
|  | IWB74922 | 7.56E-12 | 0.14 | -2.45 | 24 | 5A 699.72 |
|  | IWB24570 | 7.90E-12 | 0.17 | 4.68 | 27 | 5A 699.72 |
|  | IWB74923 | 9.91E-12 | 0.01 | -5.18 | 28 | 5A 699.72 |
|  | IWA2429 | 1.94E-10 | 1.80 | -1.45 | 25 | 5A 344.47 |
|  | IWB33010 | 2.10E-09 | 0.46 | -4.02 | 15 | 5A 742.53 |
|  | IWB10387 | 6.60E-09 | 0.28 | -1.60 | 8 | 5A 709.71 |
|  | IWB5731 | 1.23E-08 | 0.36 | 2.66 | 13 | 5A 737.3 |
|  | IWB6768 | 1.56E-08 | 0.16 | 10.72 | 10 | 5A 709.71 |
|  | IWB65779 | 2.90E-08 | 0.04 | -1.41 | 11 | 5A 709.71 |
|  | IWB53980 | 1.25E-07 | 0.02 | 1.01 | 5 | 3B 46.23 |
|  | IWB53981 | 1.98E-07 | 0.00 | -1.02 | 4 | 3B* |
|  | IWB27442 | 4.83E-07 | 0.00 | -0.13 | 7 | 5A 742.53 |
|  | IWB12585 | 5.32E-07 | 0.11 | -4.52 | 5 | 4B 335.17 |
|  | IWB9800 | 5.76E-07 | 0.32 | -1.76 | 5 | 5A 709.71 |
|  | IWB65693 | 9.59E-07 | 0.03 | 0.26 | 5 | 5A 709.71 |
|  | IWB7479 | 1.01E-06 | 0.05 | -0.20 | 6 | 2A 384.57 |
|  | Total^c^ |  | 3.10 |  |  |  |
| Starch content | | | | | | |
| Additive | IWB72111 | 6.16E-07 | 0.24 | 0.11 | 35 | 6A 156.16 |
|  | IWB34957 | 9.90E-07 | 2.87 | 0.27 | 20 | 6A 153.27 |
|  | IWB72214 | 1.83E-06 | 1.33 | -0.27 | 21 | 6A 143.43 |
|  | Total^c^ |  | 4.17 |  |  |  |
| Test weight | | | | | | |
| Additive | IWB58695 | 2.50E-06 | 10.22 | -1.06 | 17 | 2B 583.38 |
|  | Total |  | 10.13 |  |  |  |
| Dominance | IWB4196 | 1.14E-06 | 0 | -0.01 | 16 | 2B 298.27 |
|  | Total^c^ |  | 0 |  |  |  |
| 1000-kernel weight | | | | | | |
| Additive | IWB7733 | 4.09E-10 | 18.12 | -6.48 | 93 | 3B 262.85 |
|  | IWB35363 | 4.56E-10 | 0.69 | 0.18 | 78 | 3B* |
|  | IWB55272 | 5.44E-10 | 0.49 | 2.69 | 91 | 3B 262.85 |
|  | IWB62875 | 5.49E-10 | 0.10 | -0.67 | 92 | 3B 262.85 |
|  | IWB34941 | 8.55E-10 | 0.08 | -2.72 | 89 | 3B 262.85 |
|  | IWB8317 | 8.95E-10 | 0.60 | -5.03 | 89 | 3B 262.85 |
|  | IWB23272 | 3.79E-08 | 0.76 | -0.72 | 65 | 3B 255.31 |
|  | IWB41092 | 2.59E-07 | 6.70 | 0.59 | 16 | 5B* |
|  | IWB12031 | 3.83E-07 | 0.60 | -1.41 | 15 | 5B* |
|  | IWA1423 | 5.69E-07 | 3.24 | -0.78 | 28 | 6A 190.27 |
|  | IWB57308 | 1.06E-06 | 0.01 | 0.15 | 32 | 3B 255.81 |
|  | IWB57465 | 2.61E-06 | 1.70 | 0.48 | 5 | 1A 241.88 |
|  | IWB65785 | 2.80E-06 | 0.00 | 0.05 | 24 | 3B 262.18 |
|  | IWB66162 | 2.85E-06 | 0.13 | -1.55 | 31 | 3B 255.81 |
|  | IWB60904 | 2.89E-06 | 0.11 | 1.13 | 32 | 3B 256.15 |
|  | Total^c^ |  | 32.57 |  |  |  |
| Dominance | IWB9673 | 1.28E-06 | 4.22 | 0.95 | 2 | 2B 54.13 |
|  | Total^c^ |  | 4.14 |  |  |  |

^a^ “P-value” represents the original P-values before Bonferroni-Holm correction

^b^ “Frequency” represents the detection frequency of markers in 100 cross-validation runs.

^c^ “Total” represents the total proportion of genotypic variance (*p_G_*) explained by all additive markers or dominance markers detected.
