# Supplementary material for: Predicting Hybrid Performances for Quality Traits through Genomic-Assisted Approaches in Central European Wheat
Source: PLoS One. 2016 Jul 6;11(7):e0158635. doi: 10.1371/journal.pone.0158635 (PMC4934823; doi:10.1371/journal.pone.0158635)
Supplement: S3 Table — (DOCX) [file pone.0158635.s008.docx]

**S3 Table. Putative QTLs for wheat quality traits reported in previous studies.**

| Marker | Chr.^a^ | Pos.^b^ (cM) | *p_G_*^c^ (%) | Population | Literature |
| --- | --- | --- | --- | --- | --- |
| Gluten content | | | | | |
| QWgc.caas-1A | 1A |  | 4.8 | RIL | (Li et al. 2009) |
| QWgc.caas-1B | 1B |  | 4.4-5.5 | RIL | (Li et al. 2009) |
| QWgc.caas-2A | 2A |  | 6.0 | RIL | (Li et al. 2009) |
| QWgc.caas-2B.1 | 2B |  | 5.0 | RIL | (Li et al. 2009) |
| QWgc.caas-2B.2 | 2B |  | 8.8 | RIL | (Li et al. 2009) |
| QWgc.caas-2B.3 | 2B |  | 11.3 | RIL | (Li et al. 2009) |
| QWgc.caas-3A | 3A |  | 3.8-8.5 | RIL | (Li et al. 2009) |
| QWg.caas-3B | 3B |  | 3.4-6.4 | RIL | (Li et al. 2009) |
| QWgc.caas-4D.1 | 4D |  | 4.1-7.6 | RIL | (Li et al. 2009) |
| QWgc.caas-4D.2 | 4D |  | 5.1 | RIL | (Li et al. 2009) |
| QWgc.caas-5B.1 | 5B |  | 7.7 | RIL | (Li et al. 2009) |
| QWgc.caas-5B.2 | 5B |  | 5.3 | RIL | (Li et al. 2009) |
| QWgc.caas-5D | 5D |  | 7.2 | RIL | (Li et al. 2009) |
| QWgc.caas-6D | 6D |  | 4.6 | RIL | (Li et al. 2009) |
| QWgc.caas-7B | 7B |  | 4.8-5.6 | RIL | (Li et al. 2009) |
| QWgc.caas-7D | 7D |  | 10.3 | RIL | (Li et al. 2009) |
| Kernel hardness | | | | | |
| Pe-Gh-1A | 1A | 83 | 3.1 | DH | (Perretant et al. 2000) |
| Pe-Gh-5D | 5D | 1 | 66.9 | DH | (Perretant et al. 2000) |
| Pe-Gh-6D | 6D | 53 | 5.5 | DH | (Perretant et al. 2000) |
| Za-Gh-2A | 2A |  | 15-24 | RIL | (Zanetti et al. 2001) |
| Za-Gh-3B | 3B |  | 10-14 | RIL | (Zanetti et al. 2001) |
| Za-Gh-4A | 4A |  | 10-14 | RIL | (Zanetti et al. 2001) |
| Za-Gh-4D | 4D |  | 5-9 | RIL | (Zanetti et al. 2001) |
| Za-Gh-5A | 5A |  | 10-14 | RIL | (Zanetti et al. 2001) |
| Za-Gh-6B | 6B |  | 5-9 | RIL | (Zanetti et al. 2001) |
| Za-Gh-7A | 7A |  | 5-9 | RIL | (Zanetti et al. 2001) |
| Za-Gh-7B | 7B |  | 5-9 | RIL | (Zanetti et al. 2001) |
| Za-Gh-7D | 7D |  | 10-14 | RIL | (Zanetti et al. 2001) |
| Za-Gh-7D | 7D |  | 5-9 | RIL | (Zanetti et al. 2001) |
| OPB15_880_ |  |  | 8.61 | RIL | (Galande et al. 2001) |
| OPJ01_700_ |  |  | 5.97 | RIL | (Galande et al. 2001) |
| OPC16_860_ |  |  | 5.36 | RIL | (Galande et al. 2001) |
| UBC807_650_ | 6B |  | 4.79 | RIL | (Galande et al. 2001) |
| UBC873_880_ |  |  | 4.29 | RIL | (Galande et al. 2001) |
| UBC880_1000_ |  |  | 3.62 | RIL | (Galande et al. 2001) |
| OPA04_750_ |  |  | 3.26 | RIL | (Galande et al. 2001) |
| QGh.Z86-2D.a | 2D | 73.1 | 8.7 | AB | (Kunert et al. 2007) |
| QGh.Z86-5D.a | 5D | 8.9 | 13.1 | AB | (Kunert et al. 2007) |
| QGh.caas-1B | 1B |  | 27.7 | RIL | (Li et al. 2009) |
| QGh.caas-2A.1 | 2A |  | 5.4 | RIL | (Li et al. 2009) |
| QGh.caas-2A.2 | 2A |  | 3.7 | RIL | (Li et al. 2009) |
| QGh.caas-2B | 2B |  | 6.7 | RIL | (Li et al. 2009) |
| QGh.caas-2D | 2D |  | 3.5 | RIL | (Li et al. 2009) |
| QGh.caas-3A | 3A |  | 5.1 | RIL | (Li et al. 2009) |
| QGh.caas-3B | 3B |  | 5.6-7.2 | RIL | (Li et al. 2009) |
| QGh.caas-4A | 4A |  | 4.3 | RIL | (Li et al. 2009) |
| QGh.caas-4B.1 | 4B |  | 3.6-2.6 | RIL | (Li et al. 2009) |
| QGh.caas-4D.1 | 4D |  | 5.6 | RIL | (Li et al. 2009) |
| QGh.caas-4D.2 | 4D |  | 8.7 | RIL | (Li et al. 2009) |
| QGh.caas-5A | 5A |  | 8.0 | RIL | (Li et al. 2009) |
| QGh.caas-5B.1 | 5B |  | 3.8 | RIL | (Li et al. 2009) |
| QGh.caas-5B.2 | 5B |  | 5.6 | RIL | (Li et al. 2009) |
| QGh.caas-5B.3 | 5B |  | 7.0 | RIL | (Li et al. 2009) |
| QGh.caas-5D | 5D |  | 7.8 | RIL | (Li et al. 2009) |
| QGh.caas-6B | 6B |  | 2.6 | RIL | (Li et al. 2009) |
| QGh.caas-6D | 6D |  | 6.9 | RIL | (Li et al. 2009) |
| QGh.caas-7B | 7B |  | 9.4 | RIL | (Li et al. 2009) |
| QGh.caas-5D | 5D |  | 75.2 | RIL | (Zhang et al. 2009) |
| QHa.wak-2B | 2B | 78.0 | 9-16 | RIL | (Carter et al. 2012) |
| QHa.wak-2D | 2D | 56.1 | 16-21 | RIL | (Carter et al. 2012) |
| QHa.wak-4B | 4B | 0.8 | 14-15 | RIL | (Carter et al. 2012) |
| QHa.wak-6A | 6A | 38.8 | 7-8 | RIL | (Carter et al. 2012) |
| Gr-Ha-1A | 1A | 11-23-40 | 17.0 | RIL | (Groos et al. 2004) |
| Gr-Ha-4A | 4A |  | 8.1 | RIL | (Groos et al. 2004) |
| Gr-Ha-5B | 5B | 56-76-94 | 6.3 | RIL | (Groos et al. 2004) |
| Gr-Ha-6D | 6D | 63-83-118 | 6.2 | RIL | (Groos et al. 2004) |
| Gr-Ha-gwm |  |  | 12.7 | RIL | (Groos et al. 2004) |
| QHa.cob-1D | 1D | 0.0 | 8.2 | DH | (El-Feki et al. 2013) |
| QHa.cob-2B.1 | 2B | 77.8-95.4 | 9.3-16.5 | DH | (El-Feki et al. 2013) |
| QHa.cob-3B.1 | 3B | 11.80 | 5.7 | DH | (El-Feki et al. 2013) |
| QHa.cob-6B.2 | 6B | 0.0-3.0 | 7.8-12.1 | DH | (El-Feki et al. 2013) |
| QHa.cob-7A.2 | 7A | 60.0 | 9.9 | DH | (El-Feki et al. 2013) |
| QHa.cob-7D.2 | 7D | 3.0 | 9.6 | DH | (El-Feki et al. 2013) |
| Grain protein content | | | | | |
| Pe-Gpc-1B | 1B | 65 | 6.5 | DH | (Perretant et al. 2000) |
| Pe-Gpc-6A | 6A | 59 | 17.1 | DH | (Perretant et al. 2000) |
| QGpc.usw-A1 | 1A |  |  | DH | (Suprayogi et al. 2009) |
| QGpc.usw-B1 | 1B |  |  | DH | (Suprayogi et al. 2009) |
| QGpc.usw-B2 | 1B |  |  | DH | (Suprayogi et al. 2009) |
| QGpc.usw-A2 | 2A |  |  | DH | (Suprayogi et al. 2009) |
| QGpc.usw-B3 | 2B |  |  | DH | (Suprayogi et al. 2009) |
| QGpc.usw-B4 | 5B |  |  | DH | (Suprayogi et al. 2009) |
| QGpc.usw-B5 | 6B |  |  | DH | (Suprayogi et al. 2009) |
| QGpc.usw-A3 | 7A |  |  | DH | (Suprayogi et al. 2009) |
| QGpc.usw-B6 | 7B |  |  | DH | (Suprayogi et al. 2009) |
| UBC815_1000_ |  |  | 10.46 | RIL | (Galande et al. 2001) |
| OPI 20_690_ |  |  | 8.87 | RIL | (Galande et al. 2001) |
| UBC856_610_ |  |  | 8.48 | RIL | (Galande et al. 2001) |
| UBC827_1300_ |  |  | 6.4 | RIL | (Galande et al. 2001) |
| UBC807_650_ | 6B |  | 5.85 | RIL | (Galande et al. 2001) |
| OPJ01_700_ |  |  | 5.69 | RIL | (Galande et al. 2001) |
| OPB15_880_ |  |  | 5.07 | RIL | (Galande et al. 2001) |
| UBC818_1078_ |  |  | 4.46 | RIL | (Galande et al. 2001) |
| UBC812_800_ | 2B |  | 4.39 | RIL | (Galande et al. 2001) |
| UBC812_1000_ |  |  | 4.28 | RIL | (Galande et al. 2001) |
| UBC880_1200_ |  |  | 3.09 | RIL | (Galande et al. 2001) |
| QGpc.crc-2B | 2B |  | 9.9 | DH | (McCartney et al. 2006) |
| QGpc.crc-4A | 4A |  | 6.2 | DH | (McCartney et al. 2006) |
| QGpc.crc-4D | 4D |  | 29.8 | DH | (McCartney et al. 2006) |
| QGpc.crc-4D | 4D |  | 32.7 | DH | (Huang et al. 2006) |
| QGpc.crc-7B | 7B |  | 12.6 | DH | (Huang et al. 2006) |
| QGpc.B22-3A.a | 3A | 84.7 | 22.4 | AB | (Kunert et al. 2007) |
| QGpc.B22-4A.a | 4A | 12.1 | 7.3 | AB | (Kunert et al. 2007) |
| QGpc.B22-4A.b | 4A | 83.9 | 10.1 | AB | (Kunert et al. 2007) |
| QGpc.B22-5D.a | 5D | 44.0 | 4.5 | AB | (Kunert et al. 2007) |
| QGpc.B22-7B.a | 7B | 7.9 | 5.1 | AB | (Kunert et al. 2007) |
| QGpc.B22-7D.a | 7D | 66.0 | 7.8 | AB | (Kunert et al. 2007) |
| QGpc.Z86-4B.a | 4B | 35.7 | 17.6 | AB | (Kunert et al. 2007) |
| QGpc.caas-1B.1 | 1B |  | 3.2 | RIL | (Li et al. 2009) |
| QGpc.caas-1B.2 | 1B |  | 7.5 | RIL | (Li et al. 2009) |
| QGpc.caas-2A.1 | 2A |  | 6.4 | RIL | (Li et al. 2009) |
| QGpc.caas-2A.2 | 2A |  | 5.3 | RIL | (Li et al. 2009) |
| QGpc.caas-2B.1 | 2B |  | 6.4 | RIL | (Li et al. 2009) |
| QGpc.caas-2B.2 | 2B |  | 12.0 | RIL | (Li et al. 2009) |
| QGpc.caas-2D | 2D |  | 6.4 | RIL | (Li et al. 2009) |
| QGpc.caas-3A | 3A |  | 2.9-7.8 | RIL | (Li et al. 2009) |
| QGpc.caas-3B | 3B |  | 14.5 | RIL | (Li et al. 2009) |
| QGpc.caas-4D.1 | 4D |  | 3.7-14.1 | RIL | (Li et al. 2009) |
| QGpc.caas-4D.2 | 4D |  | 3.6 | RIL | (Li et al. 2009) |
| QGpc.caas-5B.1 | 5B |  | 5.3-6.3 | RIL | (Li et al. 2009) |
| QGpc.caas-5B.2 | 5B |  | 7.7 | RIL | (Li et al. 2009) |
| QGpc.caas-5D | 5D |  | 7.2 | RIL | (Li et al. 2009) |
| QGpc.caas-7B | 7B |  | 4.8-7.9 | RIL | (Li et al. 2009) |
| QGpc.caas-7D | 7D |  | 7.5 | RIL | (Li et al. 2009) |
| QGpc3A | 3A | 8.2 | 8.40 | DH | (Zhao et al. 2010) |
| QGpc3B | 3B | 1.3 | 3.31 | DH | (Zhao et al. 2010) |
| QGpc5D | 5D | 9.7 | 3.09 | DH | (Zhao et al. 2010) |
| QGpc6D | 6D | 9.6 | 3.45 | DH | (Zhao et al. 2010) |
| QGpc.mna-2B | 2B |  | 5.9-16.8 | RIL | (Tsilo et al. 2010) |
| QGpc.mna-5A | 5A |  | 6.5-11.1 | RIL | (Tsilo et al. 2010) |
| QGpc.mna-6D | 6D |  | 4.5-8.9 | RIL | (Tsilo et al. 2010) |
| wmc419 | 1B | 31.8 | 11.1 | Diversity | (Reif et al. 2011) |
| wmc18 | 2D | 64.0 | 10.3 | Diversity | (Reif et al. 2011) |
| gwm82 | 3A | 45.0 | 7.8 | Diversity | (Reif et al. 2011) |
| gwm190 | 5D | 9.0 | 4.9 | Diversity | (Reif et al. 2011) |
| QPro.wak-3B | 3B | 100.7 | 7-14 | RIL | (Carter et al. 2012) |
| Gr-Gpc-1A | 1A | 39-94-144 | 4.3-4.6 | RIL | (Groos et al. 2003) |
| Gr-Gpc-2A | 2A | 20-45-55 | 4.4-8.9 | RIL | (Groos et al. 2003) |
| Gr-Gpc-3A | 3A | 10-21-33 | 4.1-8.3 | RIL | (Groos et al. 2003) |
| Gr-Gpc-3B | 3B | 19-33-175 | 4.3-5.3 | RIL | (Groos et al. 2003) |
| Gr-Gpc-4A | 4A |  | 4.9-5.5 | RIL | (Groos et al. 2003) |
| Gr-Gpc-4D | 4D |  | 4.6-10.3 | RIL | (Groos et al. 2003) |
| Gr-Gpc-5B | 5B | 27-43-128 | 4.6 | RIL | (Groos et al. 2003) |
| Gr-Gpc-6A | 6A |  | 4.2 | RIL | (Groos et al. 2003) |
| Gr-Gpc-7A | 7A | 68-110-189 | 4.5-5.3 | RIL | (Groos et al. 2003) |
| Gr-Gpc-7D | 7D | 49-61-63 | 6.4-10.4 | RIL | (Groos et al. 2003) |
| Gr-Gpc-3A | 3A | 0-16-37 | 8.2 | RIL | (Groos et al. 2004) |
| Gr-Gpc-4D | 4D |  | 6.2 | RIL | (Groos et al. 2004) |
| Gr-Gpc-7D | 7D | 43-58-70 | 9.6 | RIL | (Groos et al. 2004) |
| QGpc.ccsu-2A.1 | 2A |  | 20.75 | RIL | (Prasad et al. 2003) |
| QGpc.ccsu-2B.1 | 2B |  | 13.39 | RIL | (Prasad et al. 2003) |
| QGpc.ccsu-2D.1 | 2D |  | 10.36-19.60 | RIL | (Prasad et al. 2003) |
| QGpc.ccsu-2D.2 | 2D |  | 2.95 | RIL | (Prasad et al. 2003) |
| QGpc.ccsu-3D.1 | 3D |  | 16.27 | RIL | (Prasad et al. 2003) |
| QGpc.ccsu-3D.2 | 3D |  | 13.99 | RIL | (Prasad et al. 2003) |
| QGpc.ccsu-4A.1 | 4A |  | 13.36 | RIL | (Prasad et al. 2003) |
| QGpc.ccsu-4A.2 | 4A |  | 8.21 | RIL | (Prasad et al. 2003) |
| QGpc.ccsu-6B.1 | 6B |  | 16.38 | RIL | (Prasad et al. 2003) |
| QGpc.ccsu-7A.1 | 7A |  | 32.44 | RIL | (Prasad et al. 2003) |
| QGpc.cob-5B.1 | 5B | 60.0-68.0 | 8.8-12.3 | DH | (El-Feki et al. 2013) |
| QGpc.cob-6A.1 | 6A | 69.0-71.0 | 8.5-9.9 | DH | (El-Feki et al. 2013) |
| QGpc.cob-6B.1 | 6B | 28.5 | 8.0 | DH | (El-Feki et al. 2013) |
| QGpc.cob-7B | 7B | 46.1-78.8 | 6.5-8.8 | DH | (El-Feki et al. 2013) |
| QGpc.cob-7D.2 | 7D | 12.0-27.4 | 5.6-7.7 | DH | (El-Feki et al. 2013) |
| Flour protein content | | | | | |
| QFpc.crc-1B | 1B |  | 6.1 | DH | (McCartney et al. 2006) |
| QFpc.crc-2B | 2B |  | 16.7 | DH | (McCartney et al. 2006) |
| QFpc.crc-4D | 4D |  | 28.7 | DH | (McCartney et al. 2006) |
| QFpc.crc-6A | 6A |  | 2.9 | DH | (McCartney et al. 2006) |
| QFpc.crc-6B | 6B |  | 9.3 | DH | (McCartney et al. 2006) |
| QFpc.crc-2D | 2D |  | 6.6 | DH | (Huang et al. 2006) |
| QFpc.crc-4D | 4D |  | 28.6 | DH | (Huang et al. 2006) |
| QFpc.crc-7B | 7B |  | 16.5 | DH | (Huang et al. 2006) |
| QFpc.caas-3A | 3A |  | 5.9 | RIL | (Zhang et al. 2009) |
| QFpc.caas-5D | 5D |  | 27.1 | RIL | (Zhang et al. 2009) |
| QFpc3A | 3A | 7.2 | 15.11 | DH | (Zhao et al. 2010) |
| QFpc5D | 5D | 11.7 | 7.51 | DH | (Zhao et al. 2010) |
| QFpc6D | 6D | 7.6 | 6.81 | DH | (Zhao et al. 2010) |
| QFpc7D | 7D | 4.6 | 1.55 | DH | (Zhao et al. 2010) |
| Za-Fpc-1B | 1B |  | 10-14 | RIL | (Zanetti et al. 2001) |
| Za-Fpc-3B | 3B |  | 5-9 | RIL | (Zanetti et al. 2001) |
| Za-Fpc-4A | 4A |  | 5-9 | RIL | (Zanetti et al. 2001) |
| Za-Fpc-5A | 5A |  | 15-24 | RIL | (Zanetti et al. 2001) |
| Za-Fpc-5B | 5B |  | 5-9 | RIL | (Zanetti et al. 2001) |
| Za-Fpc-6B | 6B |  | 5-9 | RIL | (Zanetti et al. 2001) |
| Za-Fpc-7A | 7A |  | 5-9 | RIL | (Zanetti et al. 2001) |
| Za-Fpc-7B | 7B |  | 10-14 | RIL | (Zanetti et al. 2001) |
| Za-Fpc-7D | 7D |  | 5-9 | RIL | (Zanetti et al. 2001) |
| Ku-Fpc-1B | 1B |  | 7-9 | DH | (Kuchel et al. 2006) |
| Ku-Fpc-6A | 6A |  | 7-9 | DH | (Kuchel et al. 2006) |
| Ku-Fpc-6D | 6D |  | 8-13 | DH | (Kuchel et al. 2006) |
| Ku-Fpc-7A | 7A |  | 6 | DH | (Kuchel et al. 2006) |
| Ku-Fpc-7D | 7D |  | 13 | DH | (Kuchel et al. 2006) |
| Ro-Fpc-1B | 1B |  |  | RSL | (Rousset et al. 2001) |
| Br-Fpc-2A | 2A | 15.5 | 21.3 | DH | (Breseghello et al. 2005) |
| Br-Fpc-2B | 2B | 24.4 | 14.3 | DH | (Breseghello et al. 2005) |
| Br-Fpc-4B.1 | 4B | 13.3 | 9.9 | DH | (Breseghello et al. 2005) |
| Br-Fpc-4B.2 | 4B | 18.7 | 12.3 | DH | (Breseghello et al. 2005) |
| Br-Fpc-6B | 6B | 169.0 | 18.4 | DH | (Breseghello et al. 2005) |
| SDS value | | | | | |
| Za-Sds-1B | 1B |  | >25 | RIL | (Zanetti et al. 2001) |
| Za-Sds-1D | 1D |  | 5-9 | RIL | (Zanetti et al. 2001) |
| Za-Sds-2A.1 | 2A |  | 15-24 | RIL | (Zanetti et al. 2001) |
| Za-Sds-2A.2 | 2A |  | 5-9 | RIL | (Zanetti et al. 2001) |
| Za-Sds-3A | 3A |  | 10-14 | RIL | (Zanetti et al. 2001) |
| Za-Sds-5A.1 | 5A |  | 15-24 | RIL | (Zanetti et al. 2001) |
| Za-Sds-5A.2 | 5A |  | 5-9 | RIL | (Zanetti et al. 2001) |
| Za-Sds-5B | 5B |  | 10-14 | RIL | (Zanetti et al. 2001) |
| Za-Sds-5D | 5D |  | 15-24 | RIL | (Zanetti et al. 2001) |
| QSsd.crc-1B | 1B |  | 20.6 | DH | (McCartney et al. 2006) |
| QSsd.crc-2A | 2A |  | 3.3 | DH | (McCartney et al. 2006) |
| QSsd.crc-6A | 6A |  | 5.6 | DH | (McCartney et al. 2006) |
| QSv.crc-1B | 1B |  | 14.9 | DH | (Huang et al. 2006) |
| QSv.crc-2D | 2D |  | 14.2 | DH | (Huang et al. 2006) |
| QSv.crc-5D | 5D |  | 8.8 | DH | (Huang et al. 2006) |
| QSed.B22-5D.a | 5D | 8.9 | 17.3 | AB | (Kunert et al. 2007) |
| QSed.B22-6D.a | 6D | 72.8 | 6.2 | AB | (Kunert et al. 2007) |
| QSed.Z86-1D.a | 1D | 75.4 | 32.8 | AB | (Kunert et al. 2007) |
| QSed.Z86-5D.a | 5D | 8.9 | 11.9 | AB | (Kunert et al. 2007) |
| QSsd.caas-1A | 1A |  | 12.1-15.3 | RIL | (Li et al. 2009) |
| QSsd.caas-1B.1 | 1B |  | 13.2-31.5 | RIL | (Li et al. 2009) |
| QSsd.caas-1B.2 | 1B |  | 5.8 | RIL | (Li et al. 2009) |
| QSsd.caas-1D | 1D |  | 9.0-19.3 | RIL | (Li et al. 2009) |
| QSsd.caas-2A | 2A |  | 2.2 | RIL | (Li et al. 2009) |
| QSsd.caas-2B | 2B |  | 4.4-6.1 | RIL | (Li et al. 2009) |
| QSsd.caas-2D | 2D |  | 2.7-7.5 | RIL | (Li et al. 2009) |
| QSsd.caas-3D | 3D |  | 6.2 | RIL | (Li et al. 2009) |
| QSsd.caas-4A | 4A |  | 8.1 | RIL | (Li et al. 2009) |
| QSsd.caas-5D | 5D |  | 3.7 | RIL | (Li et al. 2009) |
| QSsd.caas-6A.1 | 6A |  | 7.3-10.1 | RIL | (Li et al. 2009) |
| QSsd.caas-6A.2 | 6A |  | 3.7 | RIL | (Li et al. 2009) |
| QZs.caas-1A | 1A |  | 8.6 | RIL | (Zhang et al. 2009) |
| QZs.caas-1B | 1B |  | 19.3 | RIL | (Zhang et al. 2009) |
| QZs.caas-1D | 1D |  | 17.6 | RIL | (Zhang et al. 2009) |
| QZs.caas-3A | 3A |  | 6.4 | RIL | (Zhang et al. 2009) |
| QZs.caas-3B | 3B |  | 7.2 | RIL | (Zhang et al. 2009) |
| QZs.caas-4A | 4A |  | 4.4 | RIL | (Zhang et al. 2009) |
| sec99 | 1B | 0.0 | 3.9 | Diversity | (Reif et al. 2011) |
| wmc419 | 1B | 31.8 | 23.2 | Diversity | (Reif et al. 2011) |
| gwm312 | 2A | 74.0 | 19.5 | Diversity | (Reif et al. 2011) |
| cfd116 | 2D | 65.8 | 3.4 | Diversity | (Reif et al. 2011) |
| wmc73 | 5B | 63.0 | 8.6 | Diversity | (Reif et al. 2011) |
| QSev.wak-3B | 3B | 106.4 | 10-11 | RIL | (Carter et al. 2012) |
| Glu-A1 | 1A |  | 6.4 | RIL | (Blanco et al. 1998) |
| Gli-B1 | 1B |  | 34.3 | RIL | (Blanco et al. 1998) |
| Xmgb77 | 3A |  | 8.8 | RIL | (Blanco et al. 1998) |
| Xpsr454 | 3B |  | 6.3 | RIL | (Blanco et al. 1998) |
| Xpsr145 | 5A |  | 6.6 | RIL | (Blanco et al. 1998) |
| Xrsq | 6A |  | 14.4 | RIL | (Blanco et al. 1998) |
| Xpsr103 | 7B |  | 13.7 | RIL | (Blanco et al. 1998) |
| Ro-Sds-1B | 1B |  |  | RSL | (Rousset et al. 2001) |
| Starch content | | | | | |
| QTst.crc-1A | 1A |  | 8.7 | DH | (McCartney et al. 2006) |
| QTst.crc-1D | 1D |  | 17.7 | DH | (McCartney et al. 2006) |
| cfd72 | 1D | 50.6 | 11.1 | Diversity | (Reif et al. 2011) |
| gwm186 | 5A | 62.0 | 14.7 | Diversity | (Reif et al. 2011) |
| wmc73 | 5B | 62.5 | 3.1 | Diversity | (Reif et al. 2011) |
| Test weight | | | | | |
| UBC880_1000_ |  |  | 7.1 | RIL | (Galande et al. 2001) |
| UBC873_880_ |  |  | 6.41 | RIL | (Galande et al. 2001) |
| UBC880_1200_ |  |  | 3.9 | RIL | (Galande et al. 2001) |
| QTw.crc-2D | 2D |  | 5.1 | DH | (Huang et al. 2006) |
| QTw.crc-4A | 4A |  | 5.4 | DH | (Huang et al. 2006) |
| QTw.crc-4D | 4D |  | 13.1 | DH | (Huang et al. 2006) |
| QTw.crc-5A | 5A |  | 8.5 | DH | (Huang et al. 2006) |
| QTw.crc-7A | 7A |  | 10.6 | DH | (Huang et al. 2006) |
| QHlw.B22–3B.a | 3B | 60.9 | 6.0 | AB | (Kunert et al. 2007) |
| QHlw.B22-4A.a | 4A | 83.9 | 8.3 | AB | (Kunert et al. 2007) |
| QHlw.B22-6B.a | 6B | 47.7 | 7.9 | AB | (Kunert et al. 2007) |
| QHlw.B22-7A.a | 7A | 72.2 | 12.1 | AB | (Kunert et al. 2007) |
| QHlw.B22-7A.b | 7A | 99.8 | 7.5 | AB | (Kunert et al. 2007) |
| QHlw.B22-7B.a | 7B | 53.5 | 17.8 | AB | (Kunert et al. 2007) |
| QHlw.Z86-3B.a | 3B | 11.8 | 7.8 | AB | (Kunert et al. 2007) |
| QHlw.Z86-3B.b | 3B | 66.3 | 24.3 | AB | (Kunert et al. 2007) |
| QHlw.Z86-6B.a | 6B | 55.3 | 17.2 | AB | (Kunert et al. 2007) |
| QTw.caas-4B | 4B |  | 6.9 | RIL | (Zhang et al. 2009) |
| QTw.caas-5B | 5B |  | 5.8 | RIL | (Zhang et al. 2009) |
| QTw.caas-5D | 5D |  | 6.3 | RIL | (Zhang et al. 2009) |
| QTw.caas-6D | 6D |  | 9.6 | RIL | (Zhang et al. 2009) |
| wmc336 | 1A | 35.0 | 0.7 | Diversity | (Reif et al. 2011) |
| taglgap | 1B | 0.0 | 2.0 | Diversity | (Reif et al. 2011) |
| barc149 | 1D | 14.0 | 4.1 | Diversity | (Reif et al. 2011) |
| wmc11 | 3A | 0.0 | 0.9 | Diversity | (Reif et al. 2011) |
| barc84 | 3B | 97.0 | 1.1 | Diversity | (Reif et al. 2011) |
| barc71 | 3D | 79.0 | 2.0 | Diversity | (Reif et al. 2011) |
| wmc285 | 4D | 10.0 | 3.6 | Diversity | (Reif et al. 2011) |
| wmc415 | 5A | 81.0 | 20.6 | Diversity | (Reif et al. 2011) |
| cfa2163 | 5A | 104.0 | 4.5 | Diversity | (Reif et al. 2011) |
| gwm219 | 6B | 59.0 | 34.1 | Diversity | (Reif et al. 2011) |
| barc174 | 7A | 64.0 | 18.1 | Diversity | (Reif et al. 2011) |
| gwm44 | 7D | 78.0 | 2.8 | Diversity | (Reif et al. 2011) |
| QTwt.wak-5B | 5B | 33.9 | 7-10 | RIL | (Carter et al. 2012) |
| QTw.sdau-2A | 2A |  | 12.0 | RIL | (Sun et al. 2009) |
| QTw.sdau-3B | 3B |  | 9.4 | RIL | (Sun et al. 2009) |
| QTw.sdau-4A | 4A |  | 15.3 | RIL | (Sun et al. 2009) |
| QTw.sdau-5D | 5D |  | 7.9-11.1 | RIL | (Sun et al. 2009) |
| QTw.sdau-6A | 6A |  | 14.1-18.8 | RIL | (Sun et al. 2009) |
| QTw.sdau-6B | 6B |  | 8.2-23.5 | RIL | (Sun et al. 2009) |
| QTw.sdau-7B | 7B |  | 11.3-12.3 | RIL | (Sun et al. 2009) |
| QTw.cob-1B.1 | 1B | 8.5 | 6.9 | DH | (El-Feki et al. 2013) |
| QTw.cob-6B.1 | 6B | 46.1 | 7.9 | DH | (El-Feki et al. 2013) |
| QTw.cob-7A.2 | 7A | 39.5 | 5.6 | DH | (El-Feki et al. 2013) |
| QTw.cob-7D.2 | 7D | 0.0 | 7.9 | DH | (El-Feki et al. 2013) |
| 1000-kernel weight | | | | | |
| Za-Tkw-1B | 1B |  | 10-14 | RIL | (Zanetti et al. 2001) |
| Za-Tkw-2B | 2B |  | 5-9 | RIL | (Zanetti et al. 2001) |
| Za-Tkw-3B.1 | 3B |  | 10-14 | RIL | (Zanetti et al. 2001) |
| Za-Tkw-3B.2 | 3B |  | 10-14 | RIL | (Zanetti et al. 2001) |
| Za-Tkw-5A | 5A |  | 15-24 | RIL | (Zanetti et al. 2001) |
| Za-Tkw-5B | 5B |  | 5-9 | RIL | (Zanetti et al. 2001) |
| Za-Tkw-7B.1 | 7B |  | 10-14 | RIL | (Zanetti et al. 2001) |
| Za-Tkw-7B.2 | 7B |  | 5-9 | RIL | (Zanetti et al. 2001) |
| UBC873_880_ |  |  | 6.41 | RIL | (Galande et al. 2001) |
| UBC880_1200_ |  |  | 5.83 | RIL | (Galande et al. 2001) |
| UBC856_590_ |  |  | 4.97 | RIL | (Galande et al. 2001) |
| QTgw.crc-2B | 2B |  | 6.6 | DH | (Huang et al. 2006) |
| QTgw.crc-2D | 2D |  | 9.2 | DH | (Huang et al. 2006) |
| QTgw.crc-3B | 3B |  | 3.7 | DH | (Huang et al. 2006) |
| QTgw.crc-4B | 4B |  | 6.1 | DH | (Huang et al. 2006) |
| QTgw.crc-4D | 4D |  | 26.3 | DH | (Huang et al. 2006) |
| QTgw.crc-6A | 6A |  | 13.9 | DH | (Huang et al. 2006) |
| QTgw.crc-2D.1 | 2D |  | 4.7 | DH | (Cuthbert et al. 2008) |
| QTgw.crc-2D.2 | 2D |  | 5.5 | DH | (Cuthbert et al. 2008) |
| QTgw.crc-3B.1 | 3B |  | 7.6 | DH | (Cuthbert et al. 2008) |
| QTgw.crc-3B.2 | 3B |  | 4.7 | DH | (Cuthbert et al. 2008) |
| QTgw.crc-5A | 5A |  | 10.7 | DH | (Cuthbert et al. 2008) |
| QTgw.crc-7A | 7A |  | 5.0 | DH | (Cuthbert et al. 2008) |
| QTkw.caas-1B | 1B |  | 5.8 | RIL | (Zhang et al. 2009) |
| QTkw.caas-4A | 4A |  | 8.5 | RIL | (Zhang et al. 2009) |
| QTkw.caas-5D | 5D |  | 4.6 | RIL | (Zhang et al. 2009) |
| QTkw.caas-7A | 7A |  | 6.0 | RIL | (Zhang et al. 2009) |
| wmc18 | 2D | 64.0 | 5.9 | Diversity | (Reif et al. 2011) |
| gwm160 | 4A | 79.0 | 15.0 | Diversity | (Reif et al. 2011) |
| wmc285 | 4D | 10.0 | 8.1 | Diversity | (Reif et al. 2011) |
| gwm408 | 5B | 117.0 | 7.8 | Diversity | (Reif et al. 2011) |
| orw6 | 7D | 152.0 | 21.5 | Diversity | (Reif et al. 2011) |
| orw1 | 7D | 151.0 | 2.9 | Diversity | (Reif et al. 2011) |
| QKwt.wak-2B | 2B | 128.2 | 10-16 | RIL | (Carter et al. 2012) |
| Ts-Tkw-2A | 2A |  | 8.9-10.2 | RIL | (Tsilo et al. 2010) |
| Ts-Tkw-5B | 5B |  | 5.5-10.5 | RIL | (Tsilo et al. 2010) |
| Ts-Tkw-6B | 6B |  | 5.4-12.4 | RIL | (Tsilo et al. 2010) |
| Ts-Tkw-7A | 7A |  | 8.6-20.8 | RIL | (Tsilo et al. 2010) |
| QTkw.sdau-1D | 1D |  | 13.1-20.1 | RIL | (Sun et al. 2009) |
| QTkw.sdau-5D | 5D |  | 5.9-9.6 | RIL | (Sun et al. 2009) |
| QTkw.sdau-6A | 6A |  | 6.1-13.2 | RIL | (Sun et al. 2009) |
| Xwmc150b | 5A | 55 |  | Diversity | (Breseghello and Sorrells 2006) |
| Xbarc117 | 5A | 56 |  | Diversity | (Breseghello and Sorrells 2006) |
| Xbarc308 | 5B | 129 |  | Diversity | (Breseghello and Sorrells 2006) |
| Xbarc232 | 5B | 134 |  | Diversity | (Breseghello and Sorrells 2006) |
| Gr-Tkw-1X | 1X |  | 5.1-6.1 | RIL | (Groos et al. 2003) |
| Gr-Tkw-1D | 1D | 51-76-89 | 6.3-8.7 | RIL | (Groos et al. 2003) |
| Gr-Tkw-2B | 2B | 68-72-90 | 10.7-19.7 | RIL | (Groos et al. 2003) |
| Gr-Tkw-2D | 2D | 25-37-72 | 4.7-6.8 | RIL | (Groos et al. 2003) |
| Gr-Tkw-3A | 3A | 24-39-81 | 4.8 | RIL | (Groos et al. 2003) |
| Gr-Tkw-5B | 5B | 63-130-138 | 4.9-10.4 | RIL | (Groos et al. 2003) |
| Gr-Tkw-6A | 6A | -6-19-36 | 5.2-6.7 | RIL | (Groos et al. 2003) |
| Gr-Tkw-6D | 6D | 86-101-116 | 5.4-7.5 | RIL | (Groos et al. 2003) |
| Gr-Tkw-7A | 7A | 62-140-143 | 5.2-10.3 | RIL | (Groos et al. 2003) |
| Gr-Tkw-7D | 7D | 42-62-71 | 4.8-7.5 | RIL | (Groos et al. 2003) |
| QKw.cob-1A.1 | 1A | 70.4-85.4 | 7.4-9.3 | DH | (El-Feki et al. 2013) |
| QKw.cob-1B.1 | 1B | 37.8-45.9 | 7.1-11.7 | DH | (El-Feki et al. 2013) |
| QKw.cob-2B.1 | 2B | 78.8-83.8 | 6.1-19.1 | DH | (El-Feki et al. 2013) |
| QKw.cob-2D.2 | 2D | 40.7-43.2 | 5.5-6.2 | DH | (El-Feki et al. 2013) |
| QKw.cob-3B.1 | 3B | 11.8-14.5 | 5.6-6.9 | DH | (El-Feki et al. 2013) |
| QKw.cob-6A.1 | 6A | 71.0 | 9.6-18.5 | DH | (El-Feki et al. 2013) |
| QKw.cob-7D.2 | 7D | 3.0 | 5.8 | DH | (El-Feki et al. 2013) |

^a^ Chr. is abbreviated for chromosome.

^b^ Pos. shows the location of QTL on chromosome.

^c^ *p_G_* shows the proportion of explained genotypic variance.

Blanco A et al. (1998) Genetic mapping of sedimentation volume across environments using recombinant inbred lines of durum wheat. Plant Breeding 117:413-417. doi:10.1111/j.1439-0523.1998.tb01965.x

Breseghello F, Finney PL, Gaines C, Andrews L, Tanaka J, Penner G, Sorrells ME (2005) Genetic Loci Related to Kernel Quality Differences between a Soft and a Hard Wheat Cultivar. Crop Sci 45:1685-1695. doi:10.2135/cropsci2004.0310

Breseghello F, Sorrells ME (2006) Association Mapping of Kernel Size and Milling Quality in Wheat (Triticum aestivum L.) Cultivars. Genetics 172:1165-1177. doi:10.1534/genetics.105.044586

Carter AH, Garland-Campbell K, Morris CF, Kidwell KK (2012) Chromosomes 3B and 4D are associated with several milling and baking quality traits in a soft white spring wheat (Triticum aestivum L.) population. Theoretical and Applied Genetics 124:1079-1096

Cuthbert J, Somers D, Brûlé-Babel A, Brown PD, Crow G (2008) Molecular mapping of quantitative trait loci for yield and yield components in spring wheat (Triticum aestivum L.). Theoretical and Applied Genetics 117:595-608. doi:10.1007/s00122-008-0804-5

El-Feki WM, Byrne PF, Reid SD, Lapitan NLV, Haley SD (2013) Quantitative Trait Locus Mapping for End-Use Quality Traits in Hard Winter Wheat Under Contrasting Soil Moisture Levels. Crop Sci 53:1953-1967

Galande AA et al. (2001) Genetic analysis of kernel hardness in bread wheat using PCR-based markers. Theoretical and Applied Genetics 103:601-606. doi:10.1007/PL00002915

Groos C, Bervas E, Charmet G (2004) Genetic analysis of grain protein content, grain hardness and dough rheology in a hard×hard bread wheat progeny. Journal of Cereal Science 40:93-100

Groos C, Robert N, Bervas E, Charmet G (2003) Genetic analysis of grain protein-content, grain yield and thousand-kernel weight in bread wheat. Theoretical and Applied Genetics 106:1032-1040. doi:10.1007/s00122-002-1111-1

Huang XQ et al. (2006) Molecular detection of QTLs for agronomic and quality traits in a doubled haploid population derived from two Canadian wheats (Triticum aestivum L.). Theoretical and Applied Genetics 113:753-766. doi:10.1007/s00122-006-0346-7

Kuchel H, Langridge P, Mosionek L, Williams K, Jefferies SP (2006) The genetic control of milling yield, dough rheology and baking quality of wheat. Theoretical and Applied Genetics 112:1487-1495. doi:10.1007/s00122-006-0252-z

Kunert A, Naz A, Dedeck O, Pillen K, Léon J (2007) AB-QTL analysis in winter wheat: I. Synthetic hexaploid wheat (T. turgidum ssp. dicoccoides  × T. tauschii) as a source of favourable alleles for milling and baking quality traits. Theoretical and Applied Genetics 115:683-695. doi:10.1007/s00122-007-0600-7

Li Y, Song Y, Zhou R, Branlard G, Jia J (2009) Detection of QTLs for bread-making quality in wheat using a recombinant inbred line population. Plant Breeding 128:235-243. doi:10.1111/j.1439-0523.2008.01578.x

McCartney CA et al. (2006) QTL analysis of quality traits in the spring wheat cross RL4452 × ‘AC Domain’. Plant Breeding 125:565-575. doi:10.1111/j.1439-0523.2006.01256.x

Perretant MR et al. (2000) QTL analysis of bread-making quality in wheat using a doubled haploid population. Theoretical and Applied Genetics 100:1167-1175

Prasad M, Kumar N, Kulwal P, Röder M, Balyan H, Dhaliwal H, Gupta P (2003) QTL analysis for grain protein content using SSR markers and validation studies using NILs in bread wheat. Theoretical and Applied Genetics 106:659-667. doi:10.1007/s00122-002-1114-y

Reif J et al. (2011) Association mapping for quality traits in soft winter wheat. Theoretical and Applied Genetics 122:961-970

Rousset M, Brabant P, Kota RS, Dubcovsky J, Dvorak J (2001) Use of recombinant substitution lines for gene mapping and QTL analysis of bread making quality in wheat. Euphytica 119:81-87. doi:10.1023/A:1017530002612

Sun X-Y et al. (2009) QTL analysis of kernel shape and weight using recombinant inbred lines in wheat. Euphytica 165:615-624. doi:10.1007/s10681-008-9794-2

Suprayogi Y, Pozniak C, Clarke FR, Clarke JM, Knox RE, Singh AK (2009) Identification and validation of quantitative trait loci for grain protein concentration in adapted Canadian durum wheat populations. Theoretical and Applied Genetics 119:437-448. doi:10.1007/s00122-009-1050-1

Tsilo T, Hareland G, Simsek S, Chao S, Anderson J (2010) Genome mapping of kernel characteristics in hard red spring wheat breeding lines. Theoretical and Applied Genetics 121:717-730. doi:10.1007/s00122-010-1343-4

Zanetti S, Winzeler M, Feuillet C, Keller B, Messmer M (2001) Genetic analysis of bread-making quality in wheat and spelt. Plant Breeding 120:13-19

Zhang Y et al. (2009) QTL mapping for milling, gluten quality, and flour pasting properties in a recombinant inbred line population derived from a Chinese soft × hard wheat cross. Crop and Pasture Science 60:587-597

Zhao L, Zhang K-P, Liu B, Deng Z-y, Qu H-L, Tian J-C (2010) A comparison of grain protein content QTLs and flour protein content QTLs across environments in cultivated wheat. Euphytica 174:325-335. doi:10.1007/s10681-009-0109-z
